# Immunosuppression after Sepsis: Systemic Inflammation and Sepsis Induce a Loss of Naïve T-Cells but No Enduring Cell-Autonomous Defects in T-Cell Function

**DOI:** 10.1371/journal.pone.0115094

**Published:** 2014-12-26

**Authors:** Robby Markwart, Stephanie A. Condotta, Robert P. Requardt, Farina Borken, Katja Schubert, Cynthia Weigel, Michael Bauer, Thomas S. Griffith, Martin Förster, Frank M. Brunkhorst, Vladimir P. Badovinac, Ignacio Rubio

**Affiliations:** 1 Integrated Research and Treatment Center, Center for Sepsis Control and Care (CSCC), Jena University Hospital, Jena, Germany; 2 Dept. of Pathology, University of Iowa, Iowa City, Iowa, United States of America; 3 Dept. for Anaesthesiology and Intensive Care Medicine, Jena University Hospital, Jena, Germany; 4 Minneapolis Veterans Affairs Health Care System, Minneapolis, Minnesota, United States of America; 5 Center for Immunology, University of Minnesota, Minneapolis, Minnesota, United States of America; 6 Clinic of Internal Medicine I, Jena University Hospital, Jena, Germany; 7 Center for Clinical Studies, Jena University Hospital, Jena, Germany; 8 Institute of Molecular Cell Biology, Center for Molecular Biomedicine, Jena University Hospital, Jena, Germany; University of Florida, United States of America

## Abstract

Sepsis describes the life-threatening systemic inflammatory response (SIRS) of an organism to an infection and is the leading cause of mortality on intensive care units (ICU) worldwide. An acute episode of sepsis is characterized by the extensive release of cytokines and other mediators resulting in a dysregulated immune response leading to organ damage and/or death. This initial pro-inflammatory burst often transits into a state of immune suppression characterised by loss of immune cells and T-cell dysfunction at later disease stages in sepsis survivors. However, despite these appreciations, the precise nature of the evoked defect in T-cell immunity in post-acute phases of SIRS remains unknown. Here we present an in-depth functional analysis of T-cell function in post-acute SIRS/sepsis. We document that T-cell function is not compromised on a per cell basis in experimental rodent models of infection-free SIRS (LPS or CpG) or septic peritonitis. Transgenic antigen-specific T-cells feature an unaltered cytokine response if challenged *in vivo* and *ex vivo* with cognate antigens. Isolated CD4^+^/CD8^+^ T-cells from post-acute septic animals do not exhibit defects in T-cell receptor-mediated activation at the the level of receptor-proximal signalling, activation marker upregulation or expansion. However, SIRS/sepsis induced transient lymphopenia and gave rise to an environment of immune attenuation at post acute disease stages. Thus, systemic inflammation has an acute impact on T-cell numbers and adaptive immunity, but does not cause major cell-autonomous enduring functional defects in T-cells.

## Introduction

Systemic inflammatory response syndromes (SIRS), prominently sepsis, are a leading cause of mortality in ICUs worldwide [1], [2]. Despite important advances in intensive care support and infection medicine, the burden of sepsis has not receded in recent times owing to a continuously increasing incidence as a result of an ageing population, a steady rise in surgical interventions and the surge of antibiotic resistances [3], [4]. This alarming development is aggravated by the sobering fact that significant improvements in public and academic awareness and a sepsis research boost have not translated to groundbreaking new therapies in the clinical setting [5].

By definition, sepsis describes cases of SIRS with a documented microbial infection, with further severity-based categorization into sepsis, severe sepsis or septic shock being contingent on other clinical determinants [6]. SIRS, including sepsis, share as a common initiating event the release and systemic spread of pro-inflammatory cytokines and other types of mediators in response to mostly focalised insults like trauma, burns or microbial infections. The acute, profuse release of pro-inflammatory agents, often referred to as “cytokine storm”, is thought to lie at the root of SIRS/sepsis and to be the kick-start event for a plethora of ensuing perturbances including microvascular dysfunction, hemodynamic and coagulation disorders that can ultimately culminate in organ failure [7].

Sepsis is a devastating disease with an acute short-term mortality of about 70% in the case of its most fatal manifestation, septic shock [1]. At the other vertex of the disease course, sepsis survivors often suffer from multiple sequelae that dramatically affect life expectancy and quality of life [8]–[10]. One aspect that has gathered much attention recently is the possible occurrence of a protracted state of immune suppression in the post-acute sepsis patient. In line with such a scenario, patients who survive the initial, acute pro-inflammatory episode of sepsis frequently suffer from secondary infections [11], [12] or reactivation of latent viruses [13], [14], indicating that their immune system is unable to eradicate otherwise harmless or low-virulence microbial strains. Several causes and triggers for the observed suppression of adaptive immunity in sepsis have been put forward. In 1997 Hotchkiss and co-workers presented the first in a series of studies reporting widespread loss of lymphocytes in animal models of acute sepsis [15]. Lymphocyte loss was later confirmed in sepsis patients and affected B- and T-cells similarly [16]–[18]. CD4+ T-lymphocytes, a population of special relevance for acute survival in sepsis according to some [19] but not all studies [20] was particularly vulnerable to apoptotic death in polymicrobial sepsis models [16]. T-cells succumbed to apoptosis [21] with caspase inhibitors [22], promotion of pro-survival signalling [23]–[25] or genetic ablation of pro-apoptotic factors [26] exerting protection to varying degrees in distinct experimental models. However, other modes of death like e.g. necrosis or pyroptosis of hematopoetic precursors may contribute to leukopenia in particular SIRS scenarios [27], [28].

Beyond the blow provided by the widespread death of lymphocytes other factors like e.g. a significant loss in antigen presentation capacity have been linked to compromised T-cell immunity in post-acute sepsis [29], [30]. Moreover, T-cells collected from deceased sepsis patients manifest signs of exhaustion, as they exhibit reduced cytokine production and expansion in response to surrogate antigen stimulation TCR antibodies [31], [32]. T-lymphocytes from patients in the acute phase of sepsis accumulate genuine inhibitory cell surface co-receptors such as CTLA-4 or PD-1 [33], [34], providing one rationale explanation for the impaired functional responses to antigen and arguing for a T-cell-intrinsic origin of the observed immune paralysis. Finally, evidence has recently accumulated pointing to an important role of inhibitory immune cells, including regulatory T-cells (T_reg_) and myeloid derived suppressor cells for the onset of immune suppression in SIRS/sepsis [35]–[39].

These and more related findings have shaped the notion that a selective and adequately graded boost of the adaptive immune system can be a useful strategy for the treatment of sepsis patients in the post-acute phase of the disease. However, the multiple suspected origins and causes of immune suppression render it difficult to discriminate appropriate targets for intervention. Moreover, since the majority of studies have focused on the acute phase of sepsis, the situation at post-acute stages, i.e. in patients who survived sepsis and might profit most from an immune modulatory therapy, is not well delineated. In particular, it is difficult to judge from the available stock of data whether the protracted paralysis in adaptive immunity reflects intrinsic defects in the ability of lymphocytes to react to antigen challenge or rather results from systemic perturbations that invariably accompany an episode of sepsis. We have conducted the present study employing rodent SIRS/sepsis models to understand whether or not systemic inflammation induces an enduring T-cell dysfunction that can contribute to a state of immune suppression in the post acute sepsis patient.

## Materials and Methods

### Materials, reagents and peptides

Golgi Plug Protein Transport Inhibitor, Cytofix/Cytoperm Solution, PermWash buffer and 70 µm cell strainer were purchased from BD Pharmingen (Franklin Lakes, USA). Click.iT EdU flow cytometry assay kit and sytox AADvanced from Life Technologies Corporation (Carlsbad, USA). Murine MicroBeads CD4 (L3T4), CD8α (Ly2) and the CD4/CD8 T-cell activation/Expansion Kit mouse were purchased from Miltenyi Biotec GmbH (Bergisch Gladbach, Germany). LPS (L2880) were purchased from Sigma-Aldrich (St.Louis, USA). Vetbond tissue adhesive from 3 M (St. Paul, USA). EDTA-capillary and Li-Heparin-capillary tubes were acquired from Sarstedt (Nümbrecht, Germany). RPMI 1640 medium from Biochrome AG (Berlin, Germany). FCS from Biowest LLC (Kansas City, USA). 2-Mercapto-ethanol from Roth (Karlsruhe, Germany). VitaLyse Lysing Buffer from BioE (St. Paul, USA). Streptavidin from Dianova (Hamburg, Germany). 24-well plates from Corning Incorporated (Corning, USA). CFSE from Enzo Lifescience Inc. (Lörrach, Germany). Lauryl-maltoside (n-Dodecyl-ß-D-maltoside, ULTROL Grade) from Calbiochem (Bad Soden, Germany). CpG (TCCATGACGTTCCTGACGTT) was acquired from Sigma-Aldrich (St.Louis, USA). GP33–41 (KAVYNFATM) and GP61–80 (GLKGPDIYKGVYQFKSVEFD) peptides were synthesized by Bio-Synthesis (Louisville, USA).

### Antibodies

Antibodies for flow cytometry analysis: anti-mouse fluorescein isothiocyanate (FITC)-conjugated CD3ε(clone 145-2C11), anti-mouse phycoerythrin (PE)-conjugated CD4 (clone YTS 191.1.2 and clone PJP6) and anti-mouse allophycocyanin (APC)-conjugated CD8α (clone YTS169.4) were purchased from ImmunoTools (Friesoythe, Germany). Anti-mouse PE-conjugated CD154 (clone MR1), anti-mouse APC-conjugated CD25 (clone 7D4) and anti-mouse FITC- or PE conjugated CD69 (clone H1.2F3) were purchased from Miltenyi Biotec GmbH (Bergisch Gladbach, Germany). Anti-mouse peridinin cholorphyll A protein (PerCP)-eFluor710-conjugated CD4 (clone GK1.5), anti-mouse APC-eFlour780-conjugated CD8 (clone 53-6.7), anti-mouse APC-conjugated IFNγ (clone XMG1.2), anti-mouse FITC- or PE-conjugated TNFα (clone MP), anti-mouse PerCP-cyanine 5.5 (PerCP-Cy5.5)-conjugated Thy1.1 (clone HIS51) and anti-mouse FITC-conjugated Thy1.2 (clone 53-2.1) were acquired from eBioscience Inc. (San Diego, USA).

Antibodies for T-cell stimulation: Biotin Hamster Anti-Mouse CD3ε (clone 145-2C11), Biotin Hamster Anti-mouse CD28 (clone 37.51), purified Hamster Anti-mouse CD28 (clone 37.51) and purified Hamster Anti-mouse CD3ε (clone 145-2C11) were purchased from BD Pharmingen (Franklin Lakes, USA).

Antibody for ELISA: Mouse IL-2 ELISA Max Standard Sets (BioLegend Inc., San Diego, USA).

Antibodies for Western blot analysis: P44/42 MAPK (Erk1/2) (137F5) rabbit mAb (#4695), Phospho-p44/42 MAPK (Erk1/2) (Thr202/Tyr204) rabbit mAb (#4370), Phospho-ZAP-70 (Tyr319)/Syk (Tyr352) (#2701) rabbit, panAkt (11E7) rabbit mAb (#4685), Phospho-Akt (Ser473) (D9E) rabbit mAb (#4060), LAT rabbit (#9166), Phospho-LAT (Tyr195) rabbit (#3584), were purchased from Cell Signaling (Danvers, USA). ZAP-70 (1E7.2) mouse (Sc-32760) was purchased from Santa Cruz Biotechnology Inc. (Dallas, USA). Peroxidase Labeled Antibody to rabbit IgG (H+L) (074-1506) and Peroxidase Labeled Antibody to Mouse IgG (H+L) (074-1806) were purchased from KPL (Gaithersburg, USA).

### Mouse strains and Ethics Statement

C57BL/6 and C57BL/6 Tg(Nr4a1-EGFP/cre) mice [40] were purchased from Charles River, Sulzfeld, Germany). C57BL/6 (Thy1.2/1.2) were purchased from the National Cancer Institute. C57BL/6 Thy1.1/1.1 or Thy1.1/1.2 P14 TCR-transgenic (TCR-Tg) mice were bred at the University of Iowa [41]–[43]. Mice were allowed to adapt to laboratory conditions for at least 4 days. Animals were maintained under artificial day-night cycles (12 h light-dark cycles; 23°C room temperature; 30%–60% environment humidity) and received a standard mouse diet and water *ad libitum*. After virus infection the animals were housed under specific pathogen-free conditions and transferred to biosafety level 2 housing. Experiments performed at the University of Jena were done in accordance with German legislation on protection of animals and with permission of the regional animal welfare committee of Thuringia (permit number TVA Reg. Nr. 02-046/11), who specifically approved this study. Experiments done at the University of Iowa followed approved University of Iowa Institutional Animal Care and Use Committee (IACUC) protocols (permit number # 1312217). The Iowa IACUC specifically approved this study.

### Bacteria and viruses

Attenuated actA-deficient *Listeria monocytogenes* (LM) expressing LCMV-derived GP33 epitope (LM-GP33) [44] and lymphocytic choriomeningitis virus (LCMV) Armstrong (LCMV-Arm) were used as described [45].

### Rodent Endotoxemia/SIRS/Sepsis Models

Four experimental murine models of SIRS/sepsis were used: (a) LPS (9–11 mg/kg body weight (b.w.)) was injected once i.p.. (b) CpG (4,5 g/kg b.w.) was injected i.p. at days 1, 3, 5 and 7. (c) Peritoneal contamination and infection (PCI): polymicrobial septic peritonitis was induced by the intraperitoneal injection of a defined volume of human stool suspension as described in [46]. The inoculum used for this study (3 µl/g b.w.) was generated from one frozen processed human stool batch that had been previously microbiologically characterised (for details see [46]). Antibiotic treatment (Meropenem, 25 mg/kg b.w., applied subcutaneously) was started 6 hours after the insult and injected once daily for 3 days. (d) Polymicrobial sepsis was induced by cecal ligation and puncture (CLP) [47], [48]. In brief, mice were anaesthetised and the abdomen was shaved and disinfected. A midline abdominal incision was made, the caecum was identified, and the distal one-third was ligated with 4–0 silk sutures. The ligated portion was punctured once using a 25-gauge needle and a small amount of cecal contents was extruded through the puncture. The caecum was returned into the abdomen and the peritoneum was closed with continuous suture. The skin was glued together with Vetbond tissue adhesive, and saline was injected for resuscitation. Bupivacaine was administered at the incision site, and flunixin meglumine was administered twice for postoperative analgesia.

Animals were scored at the predetermined time points. The clinical severity score reflects spontaneous activity, the response to exogenous stimuli and posture [46]. Owing to differing allowances from the Animal Welfare and Use committees in Thuringia and Iowa, individual experimental packages were performed with either one of the two septic peritonitis models.

### *Listeria monocytogenes* infection and intra-venial GP33 peptide injection

TCR-tg Thy1.1/1.2 P14 or Thy1.1/1.1 P14 CD8^+^ T cells (5,000/mouse) were obtained from spleens or peripheral blood of naive P14 mice and injected i.v. into naive WT C57BL/6 (Thy1.2/1.2) recipients at the indicated time points before or after induction of SIRS/sepsis. 10 days after induction of SIRS/sepsis mice were infected with LM-GP33 (1×10^∧^7 CFU, i.v.). The infection dose was verified in parallel by evaluating the number of colony forming units after plating and growing the same bacterial suspension. 7 days after LM-infection 0.5 or 5 ug synthetic GP33-41 peptide (see below) in 200 µL 0.9% saline was injected i.v. into peptide-receiving mice. 2 h later mice were sacrificed and spleens were harvested. A single cell suspension was prepared and immediately stained for flow cytometry (see LCMV infection and *ex vivo* LCMV-peptide stimulation). Following fluorochrome combinations were used: 1^st^ FITC-Thy1.2, PE-TNFα, PerCPCy5.5-Thy1.1, APC-IFNγ and APC-eFluor780-CD8; 2^nd^ FITC-Thy1.2, PE-CD69, PerCPCy5.5-Thy1.1, APC-CD25 and APC-eFluor780-CD8.

### LCMV infection and *ex vivo* LCMV-peptide stimulation

10 days post SIRS/sepsis mice were infected with the Armstrong strain of LCMV (LCMV-Arm, 2×10^5^ PFU i.p.). 8 days after LCMV-Arm infection mice were sacrificed and spleens from each analysed mouse were harvested. A single cell suspension was prepared and splenocytes were stimulated with GP33–41 (200 nM) or GP61-80 (50 µg/ml) ex vivo for 5 h at 37°C in the presence of Golgi Plug Protein Transport inhibitor. Cell-surface molecules were stained with mAb at 4°C for 30 min. Cells were washed with FACS buffer (PBS/1% FCS/0.1% NaN3), fixed and permeabilised with Cytofix/Cytoperm Solution and washed with 1x PermWash buffer followed by intracellular staining at 4°C for 30 min with the following fluorochrome panel: FITC-TNFα, APC-IFNγ, PerCP-eFluor710-CD4 and APC-eFluor780-CD8.

### Haemocytometry and clinical chemistry

Blood was withdrawn by puncturing the cheek vein and collected in Li-Heparin-anticoagulated blood tubes. White blood cells (WBC), red blood cells (RBC), haematocrit (HCT) and platelets were analysed by automated veterinary haematology (Poch-100iv-Diff; Sysmex, Leipzig, Germany). In order to monitor organ failure, plasma was obtained by centrifugation of blood samples. Plasma levels of lactate dehydrogenase (LDH), glutamic-oxaloacetic transaminase (GOT) and glutamic-pyruvic transaminase (GPT) were determined using the clinical chemistry analyser (Fuji Dri-Chem 3500i; Sysmex, Leipzig, Germany).

### T-cell purification

Murine CD4^+^ and CD8^+^ T-cells were isolated from pooled or single spleens using CD4 and CD8α MicroBeads via automated magnetic separation using the autoMACSPro system (Milteny Biotech, Bergisch Gladbach, Germany). Spleens were placed on a 70 µM cell strainer and carefully squashed with a plunger. Cells were washed with PBS/0.5% BSA/2 mM EDTA and spun down. The cell pellet was resuspended and incubated with erythrocyte lysis buffer (10 mM KHCO3; 150 mM NH4Cl; 0.1 mM EDTA) on ice for 3 min. The reaction was stopped with PBS/0.5% BSA/2 mM EDTA and followed by centrifugation. Cells were counted and instructions of the T-cell separation kits were followed and executed accordingly. Purity of CD4^+^ and CD8^+^ T-cell preparations was determined via flow cytometry and routinely exceeded 90%.

### T-cell cultivation and TCR stimulation

T-cells were cultured in RPMI 1640 medium supplemented with antibiotics, 3% (for 18 h stimulation experiments) or 10% heat-inactivated FCS + 0,00035% 2-mercapto-ethanol for longer cultivation.

TCR stimulation with soluble cross-linking Abs was accomplished by addition of 1.7 µg/mL CD3ε and/or CD28 mAb, each. For higher-order cross-linking in solution biotinylated CD3ε and/or biotinylated CD28 mAb was used at the same concentration followed by addition of 5 µg/mL streptavidin. CD3ε and CD28 Abs were mixed before administration for combined stimulations. Stimulation with CD3ε and CD28 mAb coated beads was carried out as described in the manufacturer's manual to the Miltenyi T-cell expansion Kit. For the surface immobilised stimulation cell culture plates were coated with 5 µg/ml CD3ε mAb diluted in PBS 2 h, at 37°C and 5% CO_2_, then washed directly before seeding the cells in the presence of 1.7 µg/ml soluble CD28 mAb. T-cells from Tg(Nr4a1-EGFP/cre) mice were additionally stimulated with 10 µg LPS or 10 µg CpG, respectively. For the analysis of T-cell receptor signalling (see below) T-cells were stimulated with 1.5 µg/ml CD3ε and/or CD28 mAb. The same amounts were used for the biotinylated antibodies and, additionally 7.5 µg/ml streptavidin.

### Flow cytometry

Flow cytometry of splenocyte preparations: 10 days post SIRS/sepsis spleens were harvested and 0.5–2×10^6^ cells of whole spleen single cell suspensions (after erythrocyte lysis) were used for flow cytometry. Splenocytes were washed with PBS/1% BSA and stained with appropriate Abs at 4°C for 30 min. Flow cytometry of peripheral leukocytes: 10 or 30 days post SIRS/sepsis mice were bleeded as described above. Blood samples were washed with PBS/1% BSA or PBS/1% FCS followed by erythrocyte lysis. After washing, cells were stained with FITC-CD3ε, PE-CD4 and APC-CD8 at 4°C for 30 min. Purified CD4/CD8 splenic T-cells were stimulated *ex vivo* as described in T-cell stimulation for 18 h. T-cells were washed with PBS/1% BSA and stained with the mAb panel FITC-CD69, PE-CD154 and APC-CD25 at 4°C for 30 min. Flow cytometry data were acquired using a FACS Canto II or FACS Calibur (BD PharmingenTM, Franklin Lakes, USA) and analysed with FlowJo software (TreeStar Inc., Ashland, USA).

### T-cell receptor signalling analysis

CD4^+^ and CD8^+^ T-cell isolated via automated magnetic separation (see above) were kept 1 h on ice, resuspended in RPMI/0.2% fatty acid free, endotoxin low BSA/25 mM HEPES pH 7.5 at a density of 3×10^6^ cells/ml and kept at 37°C in a water bath prior to stimulation of the TCR with cross-linking antibodies as described above. Aliquots of stimulated cells (3×10^6^ cells/point) were transferred into 1.5 ml reaction vials and spun down quickly (no longer than 20 s) in a table top centrifuge. The supernatant was discarded and the pelleted cells were lysed by addition of 0.25 ml lysis solution (50 mM HEPES pH 7.5, 100 mM NaCl, 5 mM MgCl_2_, 1 mM EGTA, 1% NP-40, 0,1% Lauryl maltoside, protease and phosphatase inhibitors) and vigorous vortexing. Lysates were placed 10 min at RT and kept on ice from there on. Extracts were cleared by centrifugation and the cleared supernatants were processed for Western Blot analysis.

### ELISA

Total protein levels of IL-2 upon *ex vivo* 48 h stimulation of T-cells with soluble CD3 plus CD28 mAb were measured in cell culture supernatants via standard ELISA methodologies.

### Proliferation assays

T-cells were labelled with 1 µM CFSE for 5 min at RT, washed with RPMI/10% FCS and seeded at a density of 10^6^ cells/ml on 24-well plates. T-cell stimulation was carried out as described above. 48 h later T-cells were harvested, stained with sytox AADvanced for live/dead cell discrimination and analyzed via flow cytometry. DNA synthesis was analyzed 46 h after stimulation of the T-cells using the Click.iT EdU flow cytometry assay kit (Life Technologies) following the manufacturer's instructions.

### Statistical Analysis

For statistical analysis data were analysed using Prism5 (GraphPad, La Jolla, USA) software. A two-tailed, Mann-Whitney U test with a confidence interval of 95% was performed to determine significances between two experimental groups (* p≤0.05, ** p≤0.01, ***p≤0.001). A One-way ANOVA with post-hoc Bonferroni analysis was performed to determine significances between more than two experimental groups (* p≤0.05, ** p≤0.01, ***p≤0.001). For survival analysis IBM SPSS Statistics (IBM, New York, USA) (19) was used. Log Rank (Mantel-Cox) was used to determine significances (* p≤0.05, ** p≤0.01, ***p≤0.001).

## Results

### Common and distinct clinical features of rodent models of SIRS and septic peritonitis

**Figure 1:**
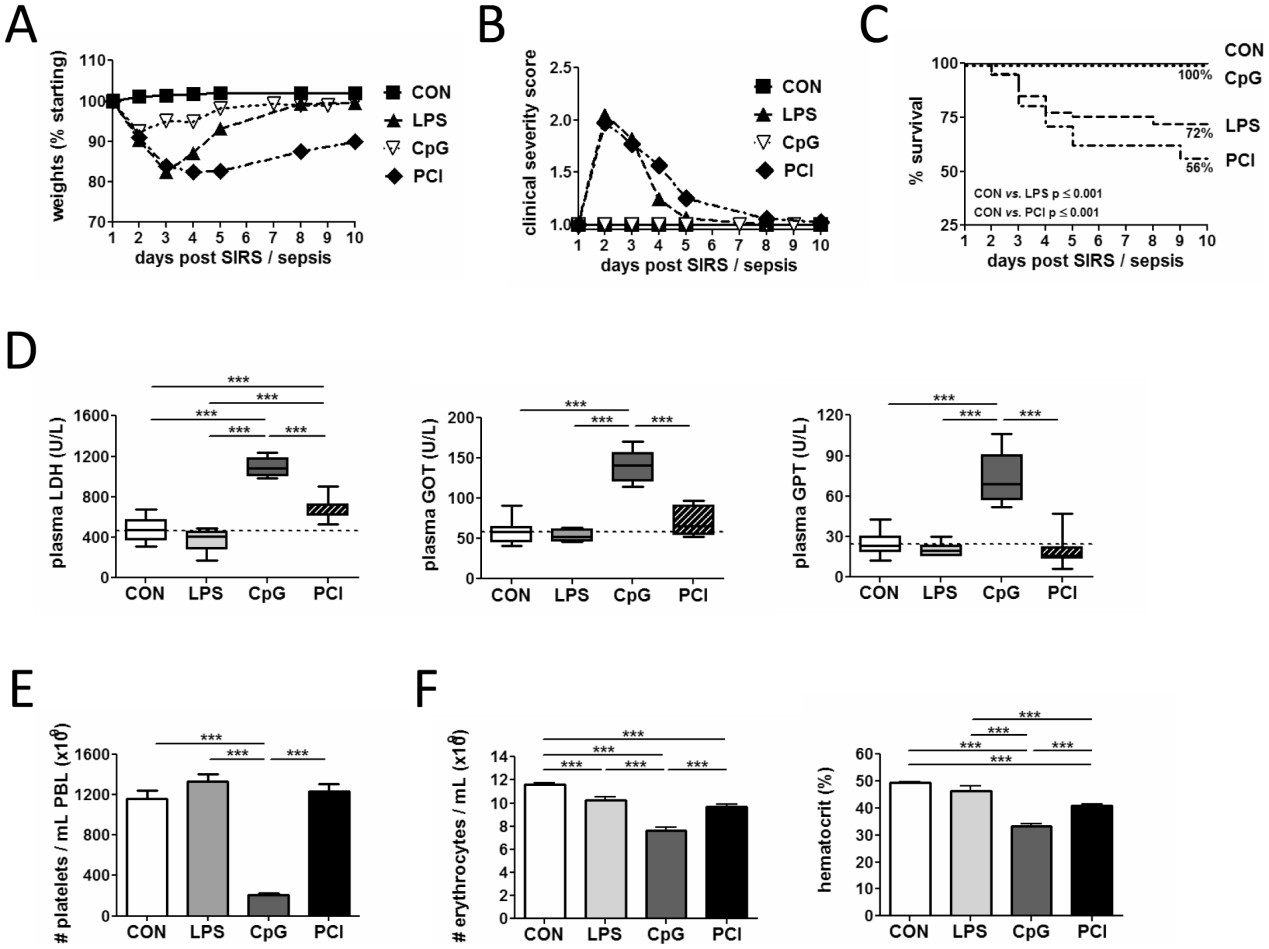
Clinical features of rodent SIRS/sepsis models. (**A**) Time course of body weight, (**B**) general morbidity and (**C**) mortality rates of mice subjected to the various SIRS/sepsis models investigated in the present study. Morbidity was evaluated using an established clinical scoring protocol [46]. Data are presented as mean + SEM including at least 26 animals per experimental group. For survival analysis Log Rank (Mantel-Cox) was used to determine significances (***p≤0.001). (**D**) Plasma levels of markers of organ damage in surviving animals 10 days after application of the insult. Data are presented as Box Whisker plots (vertical bar: median, whiskers: min and max) and include at least 5 animals per experimental group. A One-way ANOVA with post-hoc Bonferroni analysis was performed to determine significances (***p≤0.001). (**E**) Platelet numbers and (**F**) haematologic parameters in survivor animals 10 days after an episode of SIRS/sepsis. Haematocrit, platelet and erythrocyte numbers were determined via automated haemocytometry. Data are presented as mean + SEM including at least 13 animals per experimental group. A One-way ANOVA with post-hoc Bonferroni analysis was performed to determine significances (***p≤0.001). CON: healthy control.

A common hallmark of SIRS and sepsis is the early release of pro-inflammatory mediators in response to an inflammatory trigger, while only sepsis presupposes a microbial infection. To discriminate processes sparked by the cytokine storm from those dependent on the actual presence of a microbial infection and/or bacteremia we subjected mice to different protocols of infection-free SIRS or sepsis. Lipopolysaccharide or unmethylated CpG oligonucleotides act via the pattern recognition receptors TLR4 or TLR9, respectively, to elicit a pronounced systemic inflammation characterised by the rapid surge of pro-inflammatory mediators in serum [49], [50]. These two models of SIRS were compared with the effects of polymicrobial septic peritonitis induced by the intraperitoneal injection of a microbiologically characterised and calibrated suspension of human faeces (referred to as PCI, for peritoneal contamination and infection [46], [51], [52]) or, alternatively, by cecal ligation and puncture (CLP). LPS and septic peritonitis induced pronounced sickness with lethargy, weight loss, weakness and general morbidity along with high dose-dependent mortality rates (Fig. 1A-C and S1A, B Fig.), largely confirming previous reports [46], [53]–[55]. Of note, despite featuring only a modest mortality at d10 (S1B Fig.), the CLP protocol used here has been shown by us and others to induce T-cell suppression in the acute phase of sepsis [30], [35], [41], [47], [56]. Interestingly, other than a modest weight loss, none of the above morbidity traits nor lethality were observed in animals administered a cumulative 4-day CpG dose as high as 18 µg/g body weight (b.w.), despite clear signs of inflammation and organ damage (Fig. 1D). Different inter-model outcomes were also observed in platelet numbers, with animals from the LPS and PCI groups exhibiting a stabilization of platelet numbers, whilst CpG treated animals showed a prolonged, drastic 60-80% reduction in platelet numbers as late as 10 d post-insult (Fig. 1E). These observations evidenced that the three modes of SIRS varied markedly in their clinical impact despite sharing common hallmarks of systemic inflammation.

To investigate post-acute changes in T-cell function we studied T-cells at d 10 post-insult, a time point at which survivor animals had largely overcome the acute SIRS/sepsis insult as judged by the steady recovery of body weight and their clinically healthy appearance (Fig. 1A,B). A substantial degree of recovery from the acute SIRS/sepsis episode at day 10 was also illustrated by the absence of clinical markers of tissue damage (lactate dehydrogenase (LDH), glutamate pyruvate transaminase (GPT) and glutamate oxaloacetate transaminase (GOT)) in LPS treated animals (Fig. 1D), an insult that we and others have previously shown to induce substantial liver and multi-organ damage within 24 h of administration [57]. It is worth to note that animals treated with CpG or subjected to septic peritonitis still exhibited signs of organ failure at d 10, confirming our previous observations for PCI [53] and highlighting the qualitative differences between the various types of SIRS. Beyond organ failure, 10 d survivors of all groups featured further remnants of an acute systemic inflammation such as anemia, splenomegaly or bowel oedema to varying degrees (Fig. 1F, S2 Fig., and data not shown). In conclusion, the various experimental rodent models represented distinct scenarios of systemic inflammation with significant inter-model differences in mortality, morbidity and clinical parameters despite sharing the hallmarks of SIRS.

### SIRS and sepsis induce transient lymphopenia

**Figure 2:**
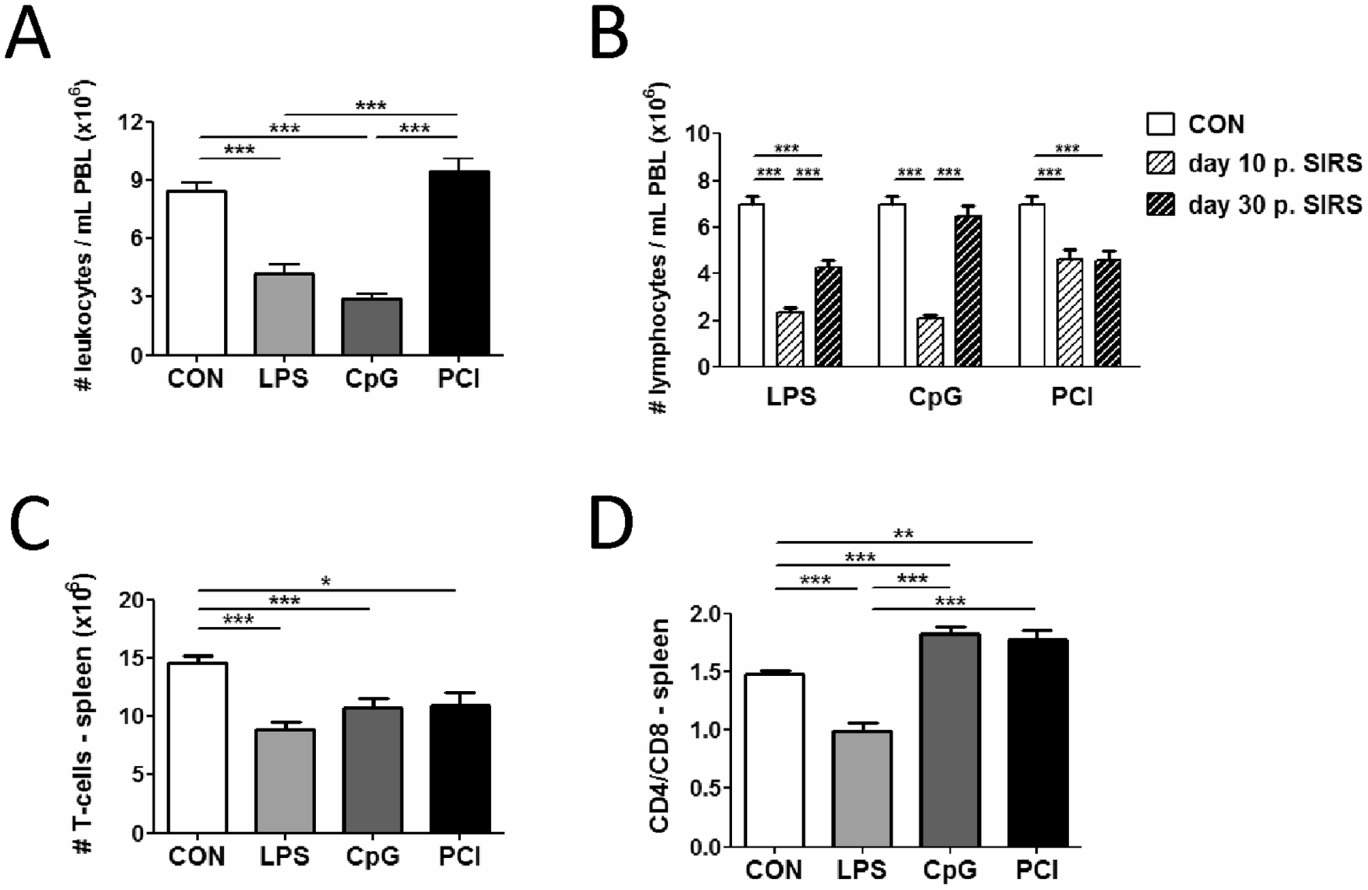
SIRS/sepsis induces lymphopenia. SIRS induced by LPS or CpG causes marked, protracted leukopenia, whilst septic peritonitis does not. (**A**) SIRS or septic peritonitis was induced in mice as described. 10 or 30 days later, white blood counts or (**B**) lymphocyte numbers in the periphery were determined by automated haemocytometry. Data are presented as mean + SEM including at least 13 animals per experimental group. A One-way ANOVA with post-hoc Bonferroni analysis was performed to determine significances (***p≤0.001). (**C**) Total T-lymphocyte counts from spleen and (**D**) CD4^+^ to CD8^+^ T-cell ratio in spleen determined in single cell preparations by flow cytometry. Data are presented as mean + SEM and include at least 9 animals per experimental group. A One-way ANOVA with post-hoc Bonferroni analysis was performed to determine significances (* p≤0.05, ** p≤0.01, ***p≤0.001). PBL: peripheral blood lymphocytes.

**Figure 3:**
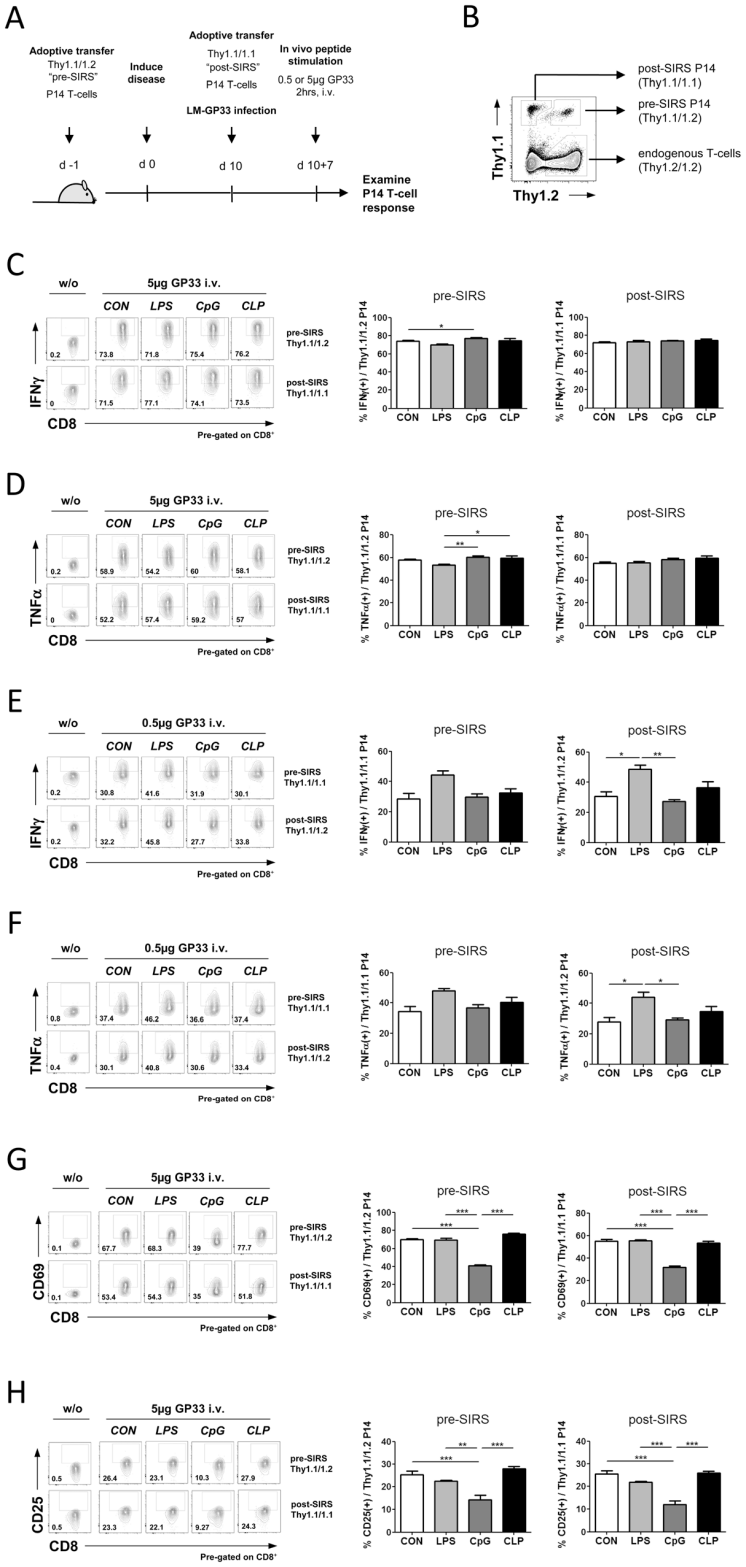
T-cell response to *in vivo* antigen challenge is not affected at post-acute stages of SIRS/sepsis. (**A**) Experimental design for *in vivo* antigenic challenge of T-lymphocytes at post-acute stages of SIRS. Mice were adoptively transferred with P14 transgenic T-lymphocytes bearing different Thy genotypes (Thy 1.1/1.2 *versus* Thy 1.1/1.1) before (pre-SIRS) and after SIRS/sepsis induction (post-SIRS). Ten days after SIRS/sepsis mice were infected with *Listeria monocytogenes* expressing the GP33 peptide. CD8^+^ T cell response was examined on day 7 post-infection 2 h after i.v. injection of 0.5 or 5 µg GP33 peptide. (**B**) Transgenic, GP33 specific CD8^+^ T-lymphocytes adoptively transferred before (pre-SIRS) or after (post-SIRS) the SIRS insult are discriminated on the basis of their different Thy genotype. (**C**) 2 h after injection of 5 µg GP33 peptide, animals were sacrificed and IFNγ or (**D**) TNFα production in CD8^+^ pre-SIRS or post-SIRS P14 T-cells was assessed by flow cytometry. (**E**) IFNγ and (**F**) TNFα production in CD8^+^ P14 T-cells after *in vivo* injection of a lower dose of 0.5 µg GP33 peptide. (**G**) Surface expression of CD69 or (**H**) CD25 was assessed by FACS analysis in the same lymphocyte populations. Data are presented as mean + SEM (4-5 mice/group [C, D, G, H], 2–4 mice/group [E, F]). A One-way ANOVA with post-hoc Bonferroni analysis was performed to determine significances (* p≤0.05, ** p≤0.01, ***p≤0.001).

**Figure 4:**
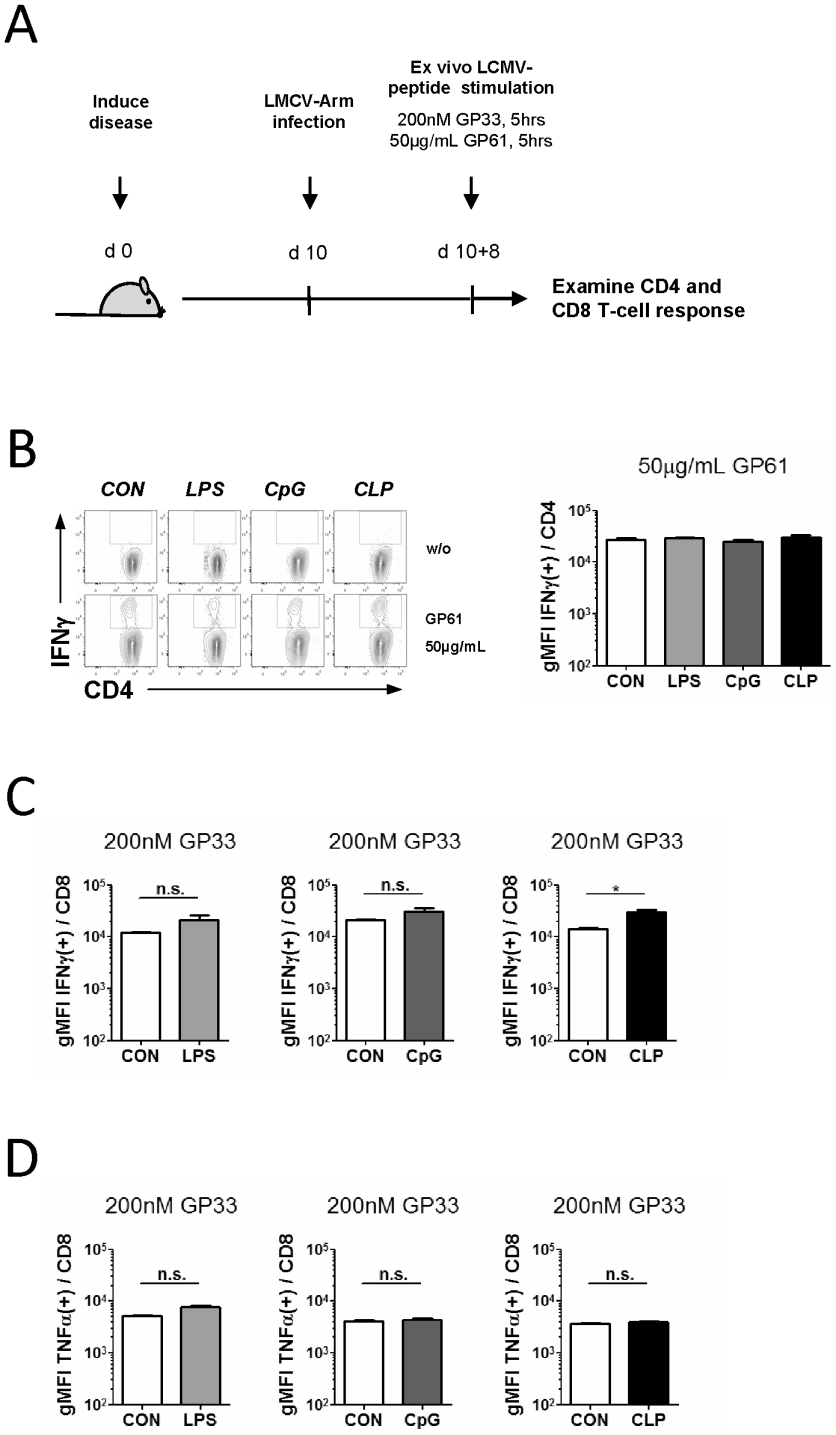
Cytokine production of CD4^+^ and CD8^+^ T-cells in response to *ex vivo* challenge with LCMV antigens is not affected at post-acute stages of SIRS/sepsis. (**A**) Experimental design. Ten days after induction of SIRS/sepsis C57BL/6 (Thy1.2/1.2) mice were infected with LCMV-Arm (2×10^5^ PFU/mouse i.p.). 8 days post infection mice were sacrificed and spleens were harvested and processed for *ex vivo* LCMV peptide stimulation. (**B**) IFNγ production in CD4^+^ T-cells following GP61 stimulation. Whole splenocyte preparations were stimulated with 50 µg/ml GP61 peptide. 5 h after stimulation the geometric mean fluorescence index (gMFI) of Ag-specific IFNγ-producing (IFNγ(+)) CD4^+^ cells was assessed via flow cytometry. (**C**) IFNγ and (**D**) TNFα production in CD8^+^ T-cells. Whole splenocyte preparations were stimulated with 200 nM GP33 LCMV peptide. 5 h after stimulation the gMFI of IFNγ-producing (IFNγ(+)) or TNFα-producing (TNFα(+)) CD8^+^ cells was assessed via flow cytometry. Data are presented as mean + SEM (3–5 mice/group) and represent two to four independent experiments. A two-tailed, Mann-Whitney U test with a confidence interval of 95% was performed to determine significances between two experimental groups. A One-way ANOVA with post-hoc Bonferroni analysis was performed to determine significances between more than two experimental groups (* p≤0.05, n.s., not significant).

The loss of T-cells as a result of widespread apoptosis in acute sepsis has been put forward as a major cause of immune paralysis [47], [58]. In agreement with that concept, the SIRS models used here induced pronounced leukopenia that was readily apparent 10 d post-insult in LPS and CpG-treated mice, but was not discernible in either of the sepsis settings (Fig. 2A and S3 Fig.). Leukopenia was largely attributable to a loss of lymphocytes, as indicated by a corresponding pattern of lymphopenia (Fig. 2B and S3 Fig.) and a drop in the numbers of splenic T-lymphocytes that was readily observed 10 d post-insult in all models, including the non-leukopenic sepsis scenario (Fig. 2C). Owing to experimental constraints (see below flowcharts in Figs. 3A and 4A and experimental section), T-cell numbers from CLP-treated animals at day 10 post surgery had to be evaluated from peripheral blood. Importantly, T-cell populations were not indiscriminately affected. Thus, animals subjected to LPS-induced endotoxemia or CLP suffered a higher fractional loss of CD4^+^ T-cells whereas CpG or PCI treated animals suffered a proportionally higher, albeit moderate, loss of CD8^+^ lymphocytes (Fig. 2D and S3 Fig.) [16], [59]. Lymphopenia was protracted as peripheral lymphocyte numbers only partially recovered between d 10 and d 30 post-insult (Fig. 2B). Of note, animals that had experienced an episode of sepsis showed only poor or no replenishment of the lymphocyte compartment in the same period of time. We conclude that lymphopenia is a common hallmark of systemic inflammation syndromes and that the ability to restore the lymphocyte compartment after SIRS or sepsis is affected to a variable extent depending on the precise clinical SIRS picture.

### T-cell response to *in vivo* antigen stimulation after SIRS or septic peritonitis

Human T-cells collected in the acute stadium of sepsis reportedly present signs of exhaustion and feature a dampened response to a TCR challenge [32] suggesting that, in addition to lymphopenia, an inherent T-cell malfunction can contribute to immune suppression in sepsis. To understand if SIRS/sepsis induces lasting defects in the functionality of T-cells beyond the acute stage of the disease we devised the two-hit experiment depicted in Fig. 3A. To identify direct consequences of the acute SIRS episode on T-cell function, mice were adoptively transferred with transgenic P14 T-lymphocytes selective for the Lymphocytic choriomeningitis virus GP33 peptide before induction of SIRS/sepsis and 10 days after the SIRS insult. P14 cells administered before (“pre-SIRS”) and after (“post-SIRS”) the acute SIRS episode were discriminated by virtue of their variant Thy1.1/1.1. or Thy1.1/1.2. genotypes (Fig. 3B, see S4 Fig. for full gating strategy), allowing for the simultaneous analysis of both transgenic P14 populations. 10 d after induction of SIRS or septic peritonitis by CLP, mice were infected with recombinant Listeria monocytogenes expressing LCMV-derived GP33 (LM-GP33) epitope to induce a GP33-specific CD8^+^ T cell response. Animals were administered 5 µg GP33 peptide i.v. to challenge P14 CD8^+^ effector T-cells 7 days after LM-GP33 infection. After 2 h the response of pre-SIRS P14 Thy 1.1/1.1 and post-SIRS P14 Thy 1.1/1.2 T-lymphocytes to the antigenic challenge was assessed in splenocyte preparations. P14 T-cells from control and all SIRS/sepsis groups reacted equally well to the *in vivo* GP33 antigenic challenge in terms of IFNγ production (Fig. 3C). This was true for both the P14 Thy 1.1./1.1 “pre-sepsis” T-cell population that had experienced the acute SIRS/sepsis episode, as well as for the P14 Thy 1.1/1.2 “post-sepsis” cells which had been adoptively transferred into the animals 10 d after SIRS/sepsis. Similar data were obtained when analysing TNFα production in the same lymphocyte compartments (Fig. 3D).

As judged by the robust fractional response of all P14 T-cells in the range of 50–75%, depending on the readout, we estimated that the i.v. dose of 5 µg GP33 used to activate effector P14 cells might be near to saturation. To avoid missing differences in lymphocyte responses at lower, non-saturating antigen dosage we repeated the experiment using a 10-fold lower antigen load. As expected, the fraction of P14 T-lymphocytes responding to a dose of 0.5 µg GP33 was lower than before, lying in the range of 30–45% of all P14 cells (Fig. 3E, F). However, similar to the situation when 10-fold higher dose of peptide was used, the cytokine response of P14 lymphocytes to 0.5 µg/g GP33 was not compromised by a prior episode of SIRS or sepsis (Fig. 3E, F). Expression of the early T-cell activation markers CD69 and CD25, was also increased in P14 T-cells in all experimental backgrounds, although in this case, a moderately weaker accumulation of surface CD69 (Fig. 3G) and CD25 (Fig. 3H) was detected in T-cells from CpG-treated animals. This observation indicated that in particular SIRS settings early steps of T-cell activation were indeed compromised in lymphocytes from post-acute SIRS. Importantly, since this dampened response was evident in both the “pre-sepsis” and “post-sepsis” P14 populations, we deduced that this shortcoming in early T-cell activation was not a consequence of the acute SIRS episode but was rather caused by resident environmental cues in the post-acute sepsis/SIRS animals. Taking all findings together we concluded that T-cells largely preserved the fundamental ability to respond to an antigenic challenge at post-acute stages of SIRS or sepsis, as judged by their unperturbed cytokine response.

### The response of T-cells from post-acute SIRS/sepsis to *ex vivo* antigen challenge

*In vivo* stimulation of T-cells via injection of antigenic peptides is a robust assay for sampling the general and fundamental ability of the adaptive immune system to mount a response to an infectious threat. Since our findings illustrated a largely unaltered response of effector T-cells to *in vivo* re-stimulation with antigen during post-acute sepsis, it followed that antigen experienced T-cells from post acute SIRS/sepsis were most likely not inherently compromised in their ability to react to an antigenic challenge. Since this concept contrasts to the reported malfunction of T-cells in acute sepsis [31], [32], we wished to underpin this notion by performing a similar two-hit experiment as before, but culminating with an *ex vivo* stimulation of endogenous effector T-cells in spleen homogenates (Fig. 4A). In this setting initial T-cell activation, effector T-cell generation and expansion proceed *in vivo* in response to LCMV infection, whilst the final antigenic stimulus is applied *ex vivo* under controlled conditions. The LCMV peptides GP61 and GP33 were used to challenge GP61- and GP33-specific effector CD4^+^ and CD8^+^ T-cells, respectively. Animals from the various SIRS/sepsis groups featured fluctuations, albeit not significant, in numbers of Ag-specific IFNγ^+^ CD4^+^ T-cells, probably reflecting the individually distinct early loss of relevant naive T-cell precursors (data not shown). Importantly and irrespective of the numerical values, a prior episode of SIRS or sepsis did not alter antigen responses of Ag-specific effector CD4^+^ T-cells on a *per cell* basis as the mean level of IFNγ production (plotted as geometric mean fluorescence index (gMFI)) in GP61-stimulated CD4^+^ T-cells was unaltered in all groups (Fig. 4B). Analogous results were obtained for CD8^+^ effector T-cells challenged with the LCMV peptide GP33 (Fig. 4C), except for the fact that we observed a significant increase in IFNγ production in CD8^+^ T-cells in animals from the peritoneal sepsis group. TNFα production was also not impaired in GP33-stimulated responsive CD8^+^ T-cells from all SIRS/sepsis groups (Fig. 4D), confirming that SIRS or sepsis did not cause persistent defects in the antigen sparked cytokine response. In sum, we concluded from these results that an episode of SIRS or sepsis did not compromise the intrinsic ability of CD4^+^ and CD8^+^ effector T-cells to respond to cognate antigens on a *per cell* basis.

### The response of na?ve T-cells from post-acute SIRS/sepsis to *ex vivo* TCR stimulation is unperturbed

While the bulk of the findings presented above argued for the absence of fundamental defects in T-cell function in post-acute sepsis, some observations like the lower CD69 and CD25 response to in vivo antigen challenge (Fig. 3G,H) argued for subtle changes in the response of T-cells to antigen. To understand if these alterations reflected a defect inherent to T-cells, we went on to investigate T-cell activation *in vitro* using isolated, highly pure native polyclonal CD4^+^/CD8^+^ lymphocyte preparations isolated 10 d post SIRS/sepsis. Productive T-cell activation requires activation of the TCR in combination with one or more co-receptors [60], as accomplished under physiological conditions by a number of cell-surface ligands on antigen-loaded APCs. Accordingly, anti-TCR and co-receptor mAb commonly employed to activate polyclonal T-cell populations via receptor clustering exert the strongest physiologically productive T-cell activation and expansion if added as a pair and surface immobilised [61], [62], probably reflecting the need for focalised activation and clustering of the TCR and co-receptors on the T-cell surface. Conversely, clustering of stimulatory mAb in solution, as achieved e.g. via secondary Ab or streptavidin/biotin-Ab conjugates induces strong proximal TCR signalling but remains unproductive in terms of T-cell physiological readouts like gene expression or expansion [63]. In light of these considerations and to avoid missing subtle changes in the functional response of T-cells after SIRS/sepsis, we set up a TCR stimulus panel to cover a wide range of physiologically productive and inadequate triggers. The panel consisted of biotinylated CD3ε and/or CD28 stimulating mAb administered in solution, either alone or in the presence of the clustering agent streptavidin, or surface-immobilised on the cell culture dish or latex beads. Pilot experiments with CD4^+^/CD8^+^ splenocytes yielded a picture that was congruent with the aforementioned predictions: as scored by up-regulation of the TCR-responsive immediate early gene Nur77 (S5A Fig.) or T-cell expansion (see below) the most potent physiological activation of T-cells was achieved with CD3ε/CD28 mAb immobilised either on dish or latex beads. Of note, the very same immobilised CD3ε/CD28 mAb induced much less proximal TCR signalling than their soluble counterparts (S5B Fig.), confirming that the quality of the stimulation (rather than net signal strength) was decisive for inducing a productive T-cell response. In sum, we concluded that the featured collection of TCR stimuli was well suited to detect deviations in TCR sensitivity and functional T-cell activation both ways to a primed or refractory state.

**Figure 5:**
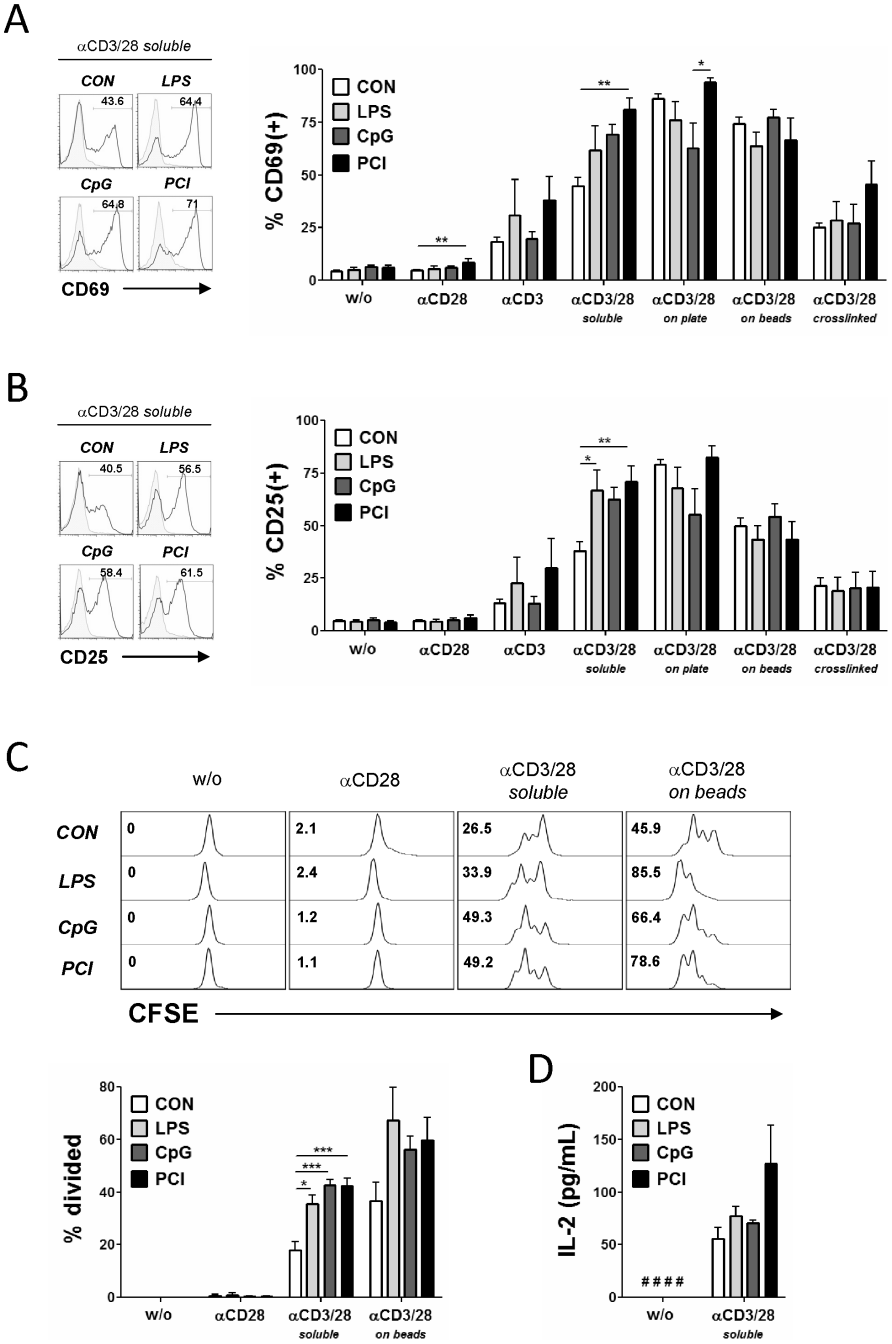
CD4^+^ and CD8^+^ T-cell response to *ex vivo* TCR challenge. (**A**) Purified CD4^+^/CD8^+^ T-cells were stimulated *ex vivo* with a panel of different TCR stimuli as described in the main text. 18 h later the surface expression of the T-cell activation marker CD69 and (**B**) CD25 was assessed by flow cytometry. Representative fluorescence profiles for stimulated (empty curves) versus non-stimulated samples (shaded curves) are shown on the left side of the panels. Data are presented as mean + SEM and represent at least four independent experiments each including at least 4 mice per group. One-way ANOVA with post-hoc Bonferroni analysis was performed for all experimental groups for each stimulation. Only statistically significant groups are labelled (* p≤0.05, ** p≤0.01). (**C**) T-cell proliferation assessed by means of CFSE dilution. T-cells from control or post-SIRS/sepsis animals were loaded with CFSE and challenged with the indicated TCR stimuli. 48 h later CFSE fluorescence was measured via flow cytometry and cell division was scored as described in the experimental section. Data are presented as mean + SEM and represent 3–4 independently processed and analysed mice. One-way ANOVA with post-hoc Bonferroni analysis was performed for all experimental groups for each stimulation. Only statistically significant groups are labelled (* p≤0.05, ** p≤0.01, ***p≤0.001). (**D**) IL2 production measured via ELISA from T-cell culture supernatants following 48 h stimulation with soluble CD3 and CD28 mAb. #: below limit of detection.

To assess if SIRS or sepsis induced enduring defects in T-cell function beyond the acute phase of the disease, T-cells isolated from spleens 10 d post-insult were challenged with this panel of TCR/co-receptor stimuli and their functional response monitored at various levels. As shown in Fig. 5A, TCR driven up-regulation of the early activation marker CD69 was virtually indistinguishable among all experimental SIRS/sepsis models and mock treated animals. Analogous results were obtained for the induction of the activation marker CD25 (Fig. 5B) and the CD4^+^ specific marker CD154 (S6A Fig.). At this stage, the only consistently observed difference was a tendentially or significantly stronger response to soluble CD3 and soluble CD3/CD28 mAb in T-cells from the SIRS/sepsis groups.

The key cell biological output to a physiologically productive TCR activation is the vigorous expansion of the challenged T-cell clones. TCR-induced proliferation of T-cells isolated from spleens 10 d post SIRS or sepsis was equal or higher than that of T-cells from controls for all used TCR stimuli as measured by CFSE dilution (Fig. 5C) or DNA synthesis (S6B Fig.). As expected, immobilised CD3/CD28 mAb generated the most productive proliferative response among tested stimuli. Interestingly, T-cells from animals that had suffered an episode of SIRS or sepsis exhibited a significantly higher proliferative score than control T-cells when challenged with soluble CD3 and CD28 Abs (Fig. 5C), reminiscent of the activation marker profiles. T-cell proliferation is driven by an autocrine signal from IL2 released by antigen-activated T-cells. In agreement with the activation marker and proliferation data, T-cells from animals with SIRS or sepsis were not impaired in the generation and release of IL-2 in response to soluble CD3 plus CD28 mAb (Fig. 5D). Taken together, these data illustrated that T-lymphocytes from animals in the post acute phase of SIRS/sepsis did not exhibit defects in their response to physiological TCR stimuli of varying strength and signalling quality.

### TCR signalling is not altered in T-cells from post acute SIRS/sepsis

While the T-cell response to stimulation with immobilised mAb was not ostensibly compromised by an episode of SIRS or sepsis, the overreaction of those very same T-cells to soluble TCR/co-receptor triggers at the level of activation marker expression and clonal expansion suggested that systemic inflammation had effectively primed and sensitised T-cells. The stronger response to soluble TCR triggers was observed irrespective of whether CD4^+^/CD8^+^ splenocytes were purified via positive or negative automated magnetic selection, arguing against an incidental consequence of an inappropriate purification strategy (unpublished observations).

**Figure 6:**
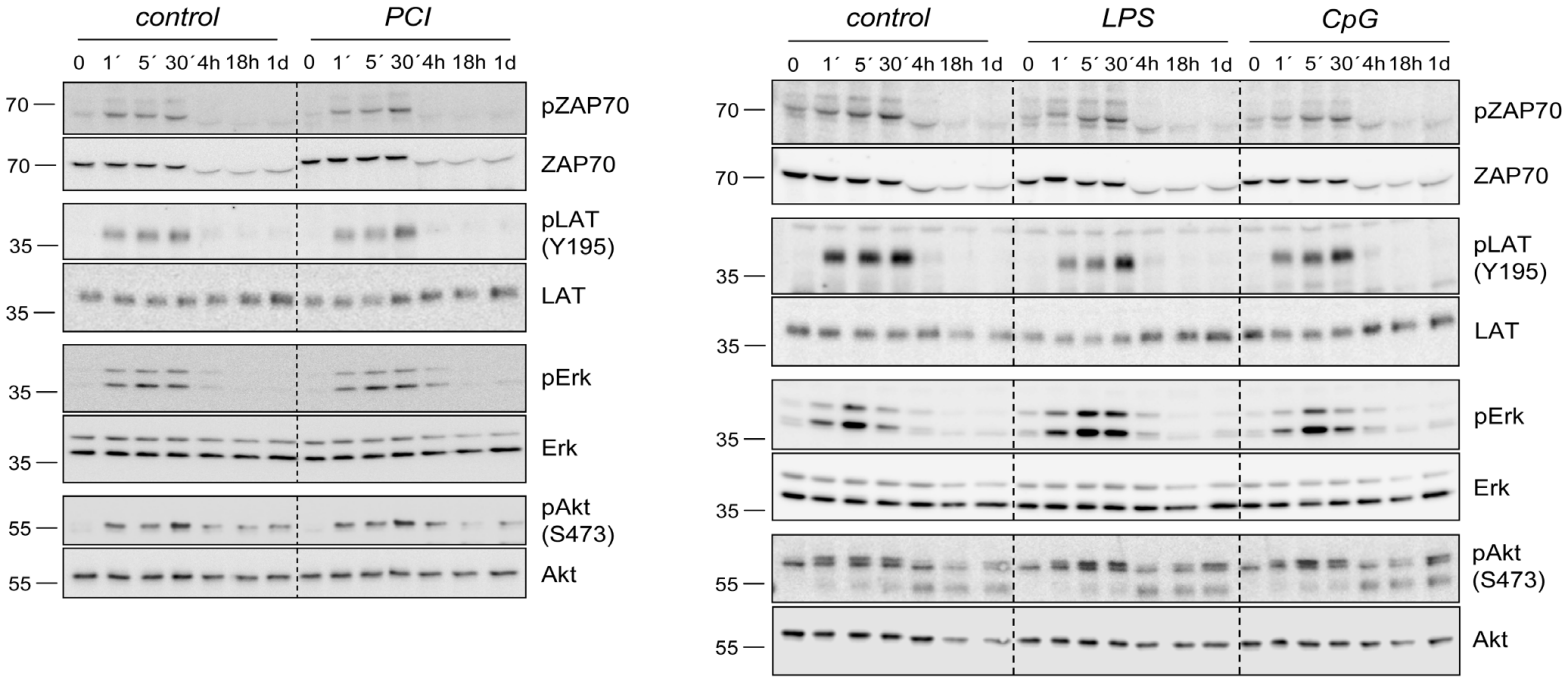
T-cells from post-acute SIRS/sepsis exhibit no alterations in proximal TCR signalling. CD4^+^/CD8^+^ splenocytes collected via automated magnetic selection 10 days after SIRS/sepsis were challenged *ex vivo* with TCR-crosslinking mAb directed against CD3 and CD28 for the indicated periods of time. Cell lysates were subjected to western blot analysis of phosphorylated and total protein levels of ZAP70, LAT, Erk and Akt. At least 2 repetitions for each of the SIRS/sepsis settings produced essentially the same results. Protein marker sizes (in kD) are given on the left side of the panel.

To understand the extent to which systemic inflammation had affected early steps of T-cell stimulation we analysed the activation status of critical signalling nodes downstream of the TCR. The acute phosphorylation/activation of ZAP-70, LAT, Erk or Akt in response to stimulation with immobilised or soluble CD3 and CD28 mAb was indistinguishable between control and any of the SIRS/sepsis groups (Fig. 6). TCR-elicited calcium transients were also not altered in T-cells from post-acute SIRS/sepsis animals (data not shown). Thus, these findings did not disclose any notable differences in acute TCR signalling that could possibly account for the observed hyper-responsiveness to soluble TCR stimuli of T-cells from post-acute SIRS/sepsis. At the same time these data confirmed and illustrated that proximal TCR signal transduction was largely intact and functional in T-cells 10 d after an episode of SIRS/sepsis.

## Discussion

Pre-clinical studies and data from animal experimentation have spurred the concept of a protracted state of immune suppression arising either alongside or as a compensatory reaction to the initial acute pro-inflammatory phase in SIRS and, more prominently, in sepsis. SIRS and sepsis describe a heterogeneous clinical picture that can manifest in multiple and variable patterns, and it is hence not difficult to assume that distinct modes of SIRS are also likely to exert individually divergent or unique effects on the host's immune system. To account for this complexity in our experimental program we employed 4 SIRS and sepsis models speculating that they would elicit both common and distinct clinical effects thus providing a broad spectrum of immunological SIRS pictures. For example, the nature of lymphopenia induced by the various SIRS and sepsis modes differed markedly, as evidenced by the gradual restoration of lymphocyte numbers in LPS or CpG induced SIRS *versus* the apparently irreparable loss of lymphocytes in the context of septic peritonitis, at least within the 30 d observational time frame investigated here. Differences in the impact of the various SIRS/sepsis models on the adaptive immune system are illustrated also by the distinct vulnerability of CD4^+^ versus CD8^+^ T-cells to any particular insult. Thus, a particularly high loss of CD4^+^ T-cells in LPS-dependent SIRS and septic peritonitis stands in contrast to a more pronounced drop of CD8^+^ T-cells in CpG-sparked SIRS. Previous studies evidenced a similar high sensitivity of rodent CD4^+^ T-cells to endotoxemia and peritonitis-borne sepsis [16], [64]. Also, patients who died of sepsis featured a proportionally higher loss of CD4^+^ T-cells [31], [65]. Available evidence on the role of CD4^+^ T-cells for onset and resolution of sepsis is controversial [19], [20]. It is worth noting at this point that whereas a contribution of innate immune cells to sepsis-linked acute mortality and morbidity is well documented, the role played by lymphocytes remains unsettled. Using knockout strains deficient in specific classes of lymphocytes some studies detected no influence of α/β T-cells on the outcome of sepsis in rodents [20], [66], others reported α/β T-cell deficient mice to be less sensitive to sepsis induced death [67], [68] and yet other studies described a protective function of T-cells in polymicrobial sepsis [19], [23], [69]. Similarly, the role played by γ/δ T-cells in onset and progression of sepsis is not clear, with studies reporting conflicting data arguing for protective [70], [71] or no marked influence of this T-cell population in sepsis survival [68]. One important aspect to be considered here is that most of the referred studies investigated the acute phase of the disease, commonly involving the observation of the first 1-3 days upon sepsis induction. Also, many reports made use of highly lethal animal models of sepsis, with 100% mortality often being reached within the first 1-3 days of disease induction and thus with no animals reaching the post-acute phase in which the adaptive immune system may, arguably, exert a more decisive influence on host survival.

Our analysis of the T-cell functions 10 d after the initiation of SIRS or sepsis aimed at closing precisely that gap in knowledge regarding the T-cell functionality in post-acute stages of sepsis. Basically, the motivation of this study was to understand whether immune suppression in post acute sepsis is a consequence of an inherent dysfunction of the T-lymphocytes. From a technical point of view it is important to stress that although our animal studies did obviously rely on the analysis of survivors, we did employ lethal to sublethal dosage in our septic peritonitis or endotoxemia models (see mortality rates in Fig. 1C). It must however be noted that despite all precautions the rodent models of SIRS and sepsis used here suffer from a number of limitations. For example, the mice are equivalent in age to young adults, a patient group which is least vulnerable to sepsis. Along these lines, aging mice are not comparably affected by co-morbidities as are elder humans. Finally, in some models, prominently CpG, the damage appears comparatively mild and it is arduous to understand to what extent it reflects severe SIRS in patients.

Irrespective of these partly inevitable drawbacks, the findings obtained in the present study were surprisingly unambiguous in the sense that they demonstrated a largely unaffected response of peripheral or splenic, na?ve or antigen experienced effector CD4^+^/CD8^+^ T-cells collected 10 d after SIRS/sepsis to a panel of TCR and co-receptor stimuli applied *in vivo* or *ex vivo* in multiple ways. Essentially all findings collected in this study indicate that T-cells from post-acute SIRS or sepsis do not carry away enduring fundamental defects in their ability to respond and react to TCR activation. It is noteworthy that the unperturbed response to *ex vivo* TCR activation was consistently observed in the background of 4 different models of SIRS/sepsis with non-overlapping clinical pictures and as adverse long-term consequences as thrombopenia (CpG), anemia (LPS, CpG, septic peritonitis), organ damage (septic peritonitis, CpG), liver failure (CpG), bacteremia (septic peritonitis) and protracted lymphopenia in all settings.

A particularly clear and streamlined outcome was obtained in experiments in which highly pure splenic polyclonal, na?ve CD4^+^/CD8^+^ T-cell preparations were challenged *ex vivo* with a panel of TCR/co-receptor antibody combinations that had been devised to sample for any irregularities in the T-cell response after SIRS/sepsis. This set of data evidenced no defects in T-cell activation at the level of proximal TCR signalling, activation marker upregulation or clonal expansion. As a matter of fact, T-cells from post acute SIRS/sepsis animals consistently exhibited a stronger response to selected, mainly soluble, TCR triggers, arguing that T-lymphocytes in post-acute SIRS/sepsis animals were possibly “primed” for an ensuing confrontation with antigen. This finding is reminiscent of the observation that trauma patients with good prognosis exhibit enhanced T-cell responses to TCR stimulation [72]. Similarly, LPS sparked endotoxemia, as the by far most widely employed experimental model of SIRS, can reportedly prime or sensitize T-cells to an antigen challenge in terms of a more robust activation/expansion [73], [74]. IL1, a potent pro-inflammatory cytokine released in high concentrations in LPS dependent endotoxemia and virtually any other setting of SIRS, is a potent inducer of na?ve T-cell responses, including expansion and cytokine release, in response to antigen challenge [75], [76]. Irrespective of what the exact mechanism of T-cell priming in post-acute SIRS may turn out to be, our findings confirm a positive priming effect of SIRS on T-cells that manifests as a more robust production of IL2, clonal expansion and cytokine response.

At first sight the observed absence of functional defects in the response of T-cells from post-acute SIRS/sepsis contrasts with previous studies that reported severe functional deficits in the response of T-lymphocytes to TCR challenge at earlier, acute stages of the disease [30], [35], [41]. For example, CD4^+^/CD8^+^ lymphocytes collected from patients deceased during acute sepsis feature reduced cytokine production and activation marker expression in response to soluble CD3/CD28 mAb mediated TCR/co-receptor stimulation [32]. Together with other findings from human sepsis patients or rodent sepsis models, including the up-regulation of well established inhibitory co-receptors such as PD-1 and CTLA-4 on T-lymphocytes [33], [34], [77], these data argued for a functional paralysis of T-lymphocytes, although an exhaustive and comprehensive characterization of T-cell functionality in acute sepsis had not yet been reported. Our observation that T-cells from post-acute phases of sepsis or SIRS are largely unaffected in their ability to respond to an antigen challenge can be reconciled in several ways with that body of data. First and most straightforward, the detected alterations in the response of T-cells collected in acute sepsis may represent transient changes in TCR sensitivity or signal processing that revert as the disease progresses. Secondly, many of the human data on T-cell functionality in acute sepsis involved the analysis of T-cell from patients who actually died from sepsis, raising the possibility of a bias in the outcome of the referred functional studies. In other words, it is conceivable that T-cells from patients who died of acute sepsis may exhibit a suppressed phenotype whereas T-cells from survivors may not. In support of this notion, epidemiological studies have provided evidence for a correlation between patient survival in trauma or sepsis and the functionality of T-cells [72], [78]. Importantly, this possibility holds valid irrespective of the still unresolved question of whether or not and how T-cells possibly contribute to sepsis eruption or to its resolution. Along this line of thinking, it is conceivable that the population of naive T-cell clones from deceased sepsis patients with a reportedly particular poor response to a TCR challenge might overlap to a significant extent with the fraction of lymphocytes that eventually succumbs to apoptosis. Since our study only included T-cells from animals that survived the acute stage of SIRS or sepsis, the findings presented in the current report are not suited to address that question, neither.

Notwithstanding the possible bias in the present and previous studies focusing uniquely either on T-cells from survivors or deceased subjects, our findings document a lack of obvious defects in T-cell activation at the post acute stage of SIRS and sepsis. It is important to stress that this conclusion should not be taken as tantamount to an absence of immune suppression. In line with a large body of literature data [16], [21], [32], [47], [79], our findings from the rodent SIRS/sepsis models do provide evidence for a compromised immunity in the surviving animals. Beyond a number of clinical manifestations of a compromised immune defence (like the occurrence and perseverance of multiple abdominal abscesses up to 30 d after septic peritonitis (data not shown)), our findings confirm the induction of pronounced lymphopenia in SIRS and sepsis, which reflects in lower numbers of antigen-specific T-cells in LCMV-Arm infected mice [47]. Based on our previous observations [80] and the data presented here we hypothesize that a loss of T-cell precursors, with the concomitant blow to the na?ve T-cell repertoire could be a major factor underlying the suppression of adaptive immunity in post acute sepsis.

Another major issue regards the possible existence of a systemic or local environment of immune attenuation in the aftermath of sepsis that could perhaps pass undetected in *ex vivo* T-cell stimulation experiments. Our data on the *in vivo* stimulation of T-cells via i.v. administration of antigenic peptides in post-SIRS animals do illustrate a moderate reduction in early T-cell activation marker upregulation in some SIRS models but no defects in the final cytokine response of T-cells. Although these experiments are instrumental in monitoring T-cell functionality and do strongly argue against major functional deficiencies of T-cells from post-acute SIRS sepsis, the value of this experimental approach as a means to sample the T-cell environment for immune attenuating cues is probably limited. For example, the systemic application of relatively high antigenic peptide doses is likely to overrun and mask rate-limiting reactions in antigen processing/presentation or other important environmental processes of T-cell activation. Thus, while the data presented herein exclude major defects in the functionality of T-cells from post-acute sepsis/SIRS, they do not provide conclusive evidence regarding the possible existence of an environment of immune attenuation in the post acute sepsis patient.

In sum, this study documents a largely unaltered response of α/ß T-cells from post-acute SIRS or sepsis to antigen/TCR stimulation indicating that T-cells are not functionally compromised on a *per cell* basis. This observation has far-reaching implications since it suggests that resident T-cells may not represent an adequate target for therapeutic intervention in the post-acute sepsis patient. This notion shifts the focus for future tentative immune stimulatory therapies to other aspects of adaptive immunity like e.g. antigen presentation [29], [30], [81] or preservation of lymphocyte clonal diversity. Indeed, the marked enduring deficiency of antigen-specific T-cells as a result of the acute and probably irreversible loss of T-cell precursors in all settings of SIRS reported here and in previous studies [47] is likely to be a major cause of immune deficiency in the post acute SIRS/sepsis patient. The reported benefit of treatments aimed at preventing early lymphocyte apoptosis in animal models of sepsis, such as the early supplementation with the pro-survival cytokines IL7 or IL15 [23], [25], [82] is consistent with this notion. A better understanding of the mechanisms and triggers of acute T-cell loss in sepsis should light the way for new avenues of therapeutic intervention aimed at preserving immunity in the post-acute sepsis patient.

## Supporting Information

S1 Fig **Mortality and morbidity in CLP-induced polymicrobial sepsis.** (**A**) Mouse body weight time plot and (**B**) mortality rates after sham or CLP surgery (at least 9 mice/group).
(TIF)

S2 Fig **Splenomegaly and splenocyte counts 10 d post SIRS/sepsis.** 10 days post SIRS/sepsis mice spleens were harvested and weighed (left panel). A single cell suspension from each analysed spleen was prepared and total numbers of splenocytes were counted with a Neubauer chamber (right panel). Data are presented as mean + SEM (at least 10 mice/group). A One-way ANOVA with post-hoc Bonferroni analysis was performed to determine significances (** p≤0.01, ***p≤0.001).
(TIF)

S3 Fig **CLP-induced polymicrobial sepsis causes lymphopenia.** 10 days after sham or CLP surgery blood was obtained and processed as described in material and methods. Total leukocyte numbers were assessed by cell counting. Total lymphocyte numbers, T-cell numbers and CD4^+^/CD8^+^ T-cell ratio were determined via flow cytometry by gating on the lymphocyte population and CD4^+^/CD8^+^ T-cells. Data are presented as mean + SEM (at least 8 mice/group). Data are representative of four independent experiments. A two-tailed, Mann-Whitney U test was performed to determine significances (n.s., not significant, ** p≤0.01, ***p≤0.001).
(TIF)

S4 Fig **Gating strategy.** Representative full gating strategy for adaptively transferred P14 T-cells. Splenic cells were identified via forward scatter (FSC)/side scatter (SSC) blotting followed by singlet gating using FSC-area (A)/FSC-width (W) blotting. Pre-SIRS P14, post-SIRS P14 and endogenous T-cells were discriminated on the basis of their different expression profile of Thy1.1 and Thy1.2 (pre-SIRS: Thy1.1/1.2; post-SIRS: Thy1.1/1.1; endogenous: Thy1.2/1.2). The percentage of IFNγ-expressing (IFNγ^+^) cells was analysed in CD8^+^ pre-SIRS and post-SIRS P14 T-cells. Gate for IFNγ^+^ P14 cells was set judged on baseline IFNγ in non challenged P14 T-cells (see Fig. 3).
(TIF)

S5 Fig **T-cell response to a panel of TCR/co-receptor Abs reflects the requirement for co-stimulation and receptor clustering.** (**A**) Splenic CD4^+^/CD8^+^ T-cells purified from transgenic C57BL/6 Tg(Nr4a1-EGFP/cre mice) (a mouse strain expressing EGFP under control of the native Nur77 promotor) were stimulated 24 h and 48 h with a panel of different TCR/co-receptor mAb combinations, 10 µg/ml LPS or 10 µg/ml CpG. EGFP expression as a readout of TCR-dependent Nur77 up-regulation was assessed by flow cytometry. Data are presented as mean + SEM and represent 3-4 independently processed and analysed mice. (**B**) CD4^+^/CD8^+^ T-cells purified from control healthy animals (CON) or from mice 10 days post SIRS/sepsis were stimulated *ex vivo* with biotinylated CD3ε and/or CD28 mAb administered in solution, either alone or in the presence of the clustering agent streptavidin (crosslinked), or surface-immobilised on latex beads. Cell lysates were subjected to western blot analysis of phosphorylated and total protein levels of Erk, ZAP70 and Akt. Depicted Western blots are representative of several independent experiments.
(TIF)

S6 Fig **The response of isolated T-cells from post-acute SIRS/sepsis to TCR activation is not compromised.** (**A**) Murine splenic CD4^+^/CD8^+^ T-cells purified magnetically 10 days after induction of SIRS/sepsis were stimulated *ex vivo* with a panel of TCR-triggers. 18 h later surface expression of the activation marker CD154 was assessed with flow cytometry. Data are presented as mean + SEM and represent at least four independent experiments each including at least 4 mice per group. There were no significant differences between experimental groups (One-way ANOVA with post-hoc Bonferroni analysis) (**B**) 48 h after stimulation DNA synthesis was assessed as a surrogate of cell proliferation by measuring the incorporation of the thymidine analogue 5-ethynyl-2′-deoxyuridine (EdU) into cellular DNA. Data are presented as mean + SEM and represent at least three independent experiments each including at least 4 mice per group. A One-way ANOVA with post-hoc Bonferroni analysis was performed to determine significances (** p≤0.01, ***p≤0.001). Only significant differences among groups are highlighted.
(TIF)
