# Supplementary figures and images for: Immunosuppression after Sepsis: Systemic Inflammation and Sepsis Induce a Loss of Naïve T-Cells but No Enduring Cell-Autonomous Defects in T-Cell Function
Source: PLoS One. 2014 Dec 26;9(12):e115094. doi: 10.1371/journal.pone.0115094 (PMC4277344; doi:10.1371/journal.pone.0115094)

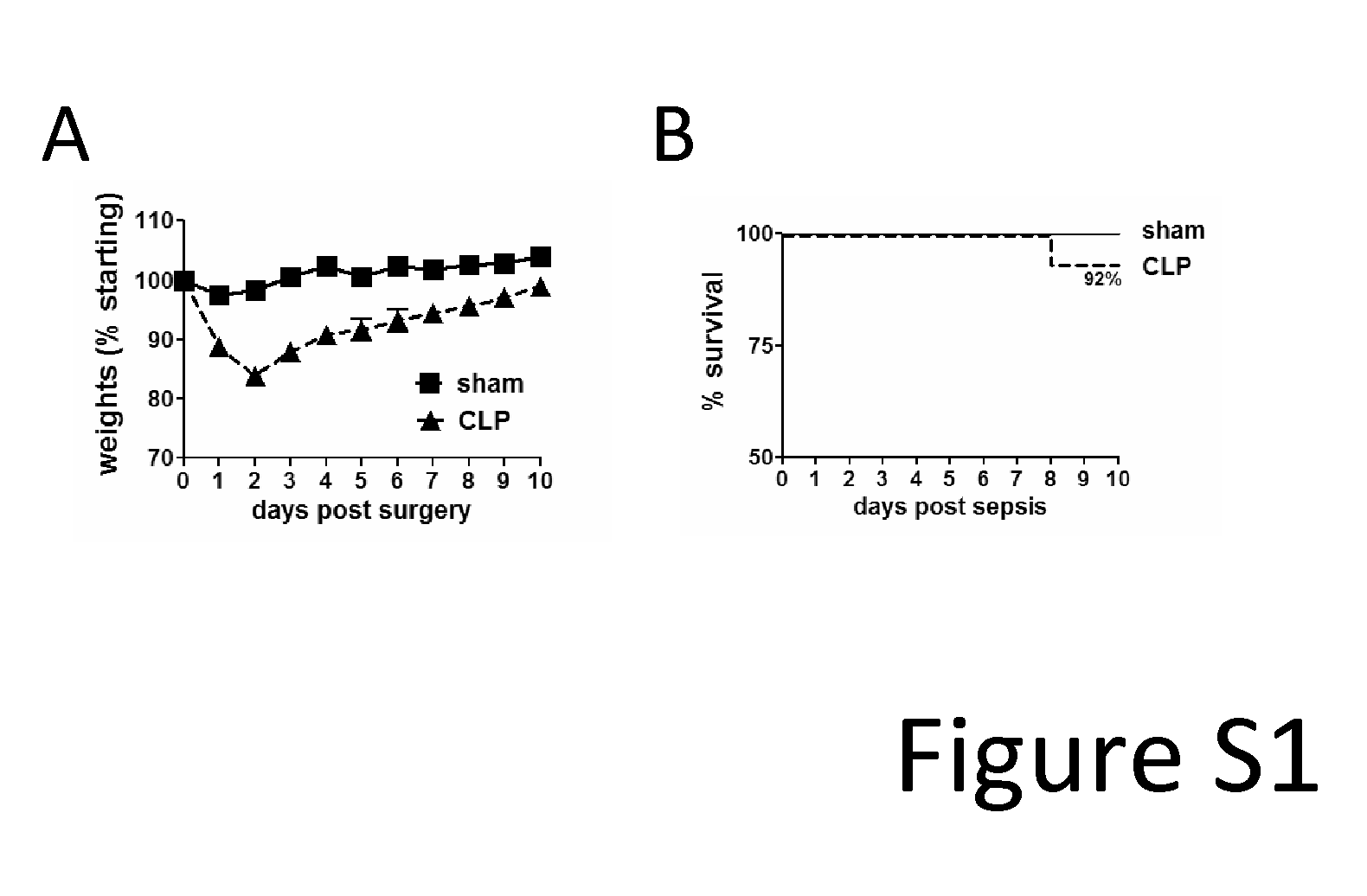

Supplement: S1 Fig — Mortality and morbidity in CLP-induced polymicrobial sepsis. (A) Mouse body weight time plot and (B) mortality rates after sham or CLP surgery (at least 9 mice/group). (TIF) [file pone.0115094.s001.tif]

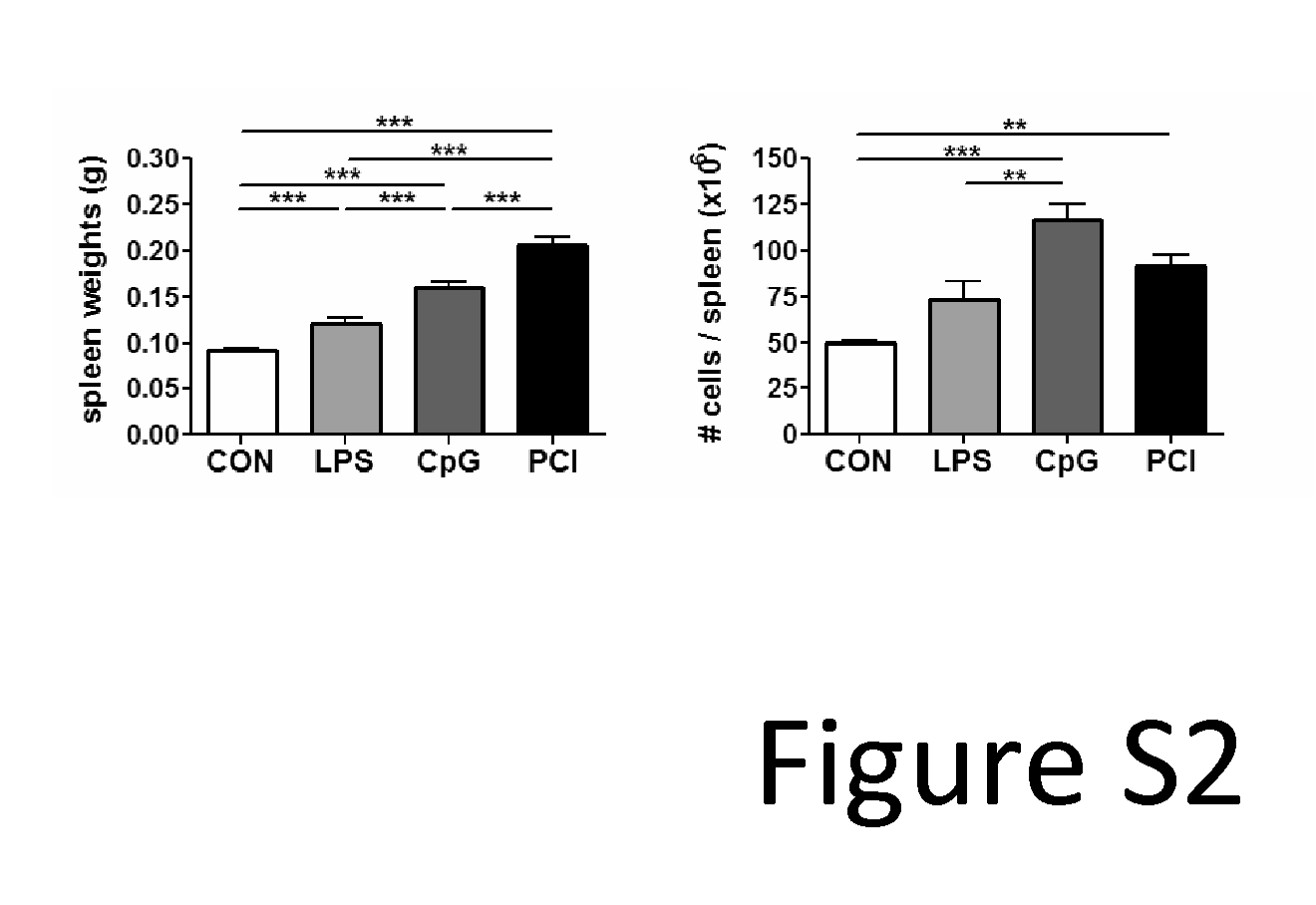

Supplement: S2 Fig — Splenomegaly and splenocyte counts 10 d post SIRS/sepsis. 10 days post SIRS/sepsis mice spleens were harvested and weighed (left panel). A single cell suspension from each analysed spleen was prepared and total numbers of splenocytes were counted with a Neubauer chamber (right panel). Data are presented as mean + SEM (at least 10 mice/group). A One-way ANOVA with post-hoc Bonferroni analysis was performed to determine significances (** p≤0.01, ***p≤0.001). (TIF) [file pone.0115094.s002.tif]

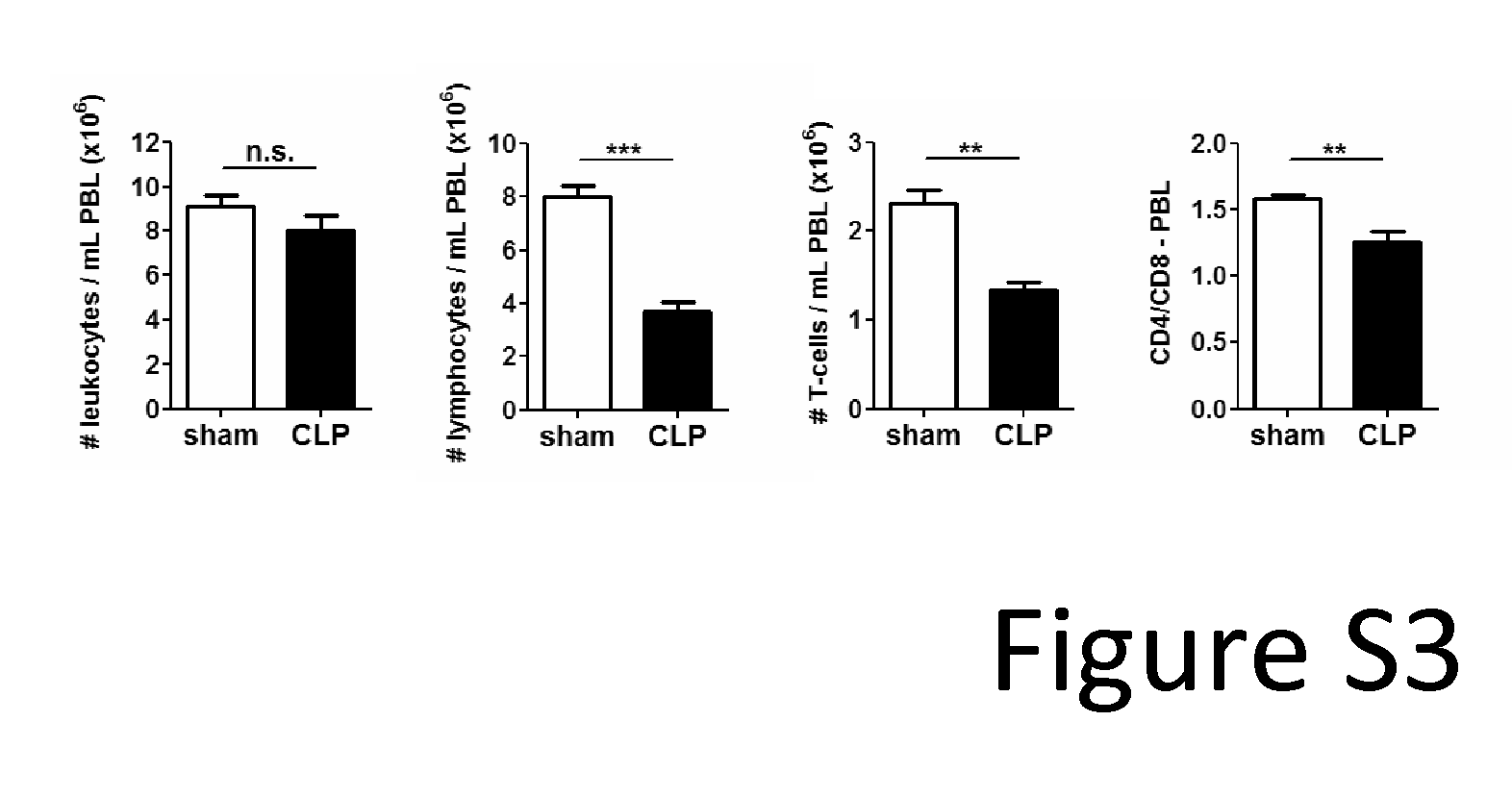

Supplement: S3 Fig — CLP-induced polymicrobial sepsis causes lymphopenia. 10 days after sham or CLP surgery blood was obtained and processed as described in material and methods. Total leukocyte numbers were assessed by cell counting. Total lymphocyte numbers, T-cell numbers and CD4+/CD8+ T-cell ratio were determined via flow cytometry by gating on the lymphocyte population and CD4+/CD8+ T-cells. Data are presented as mean + SEM (at least 8 mice/group). Data are representative of four independent experiments. A two-tailed, Mann-Whitney U test was performed to determine significances (n.s., not significant, ** p≤0.01, ***p≤0.001). (TIF) [file pone.0115094.s003.tif]

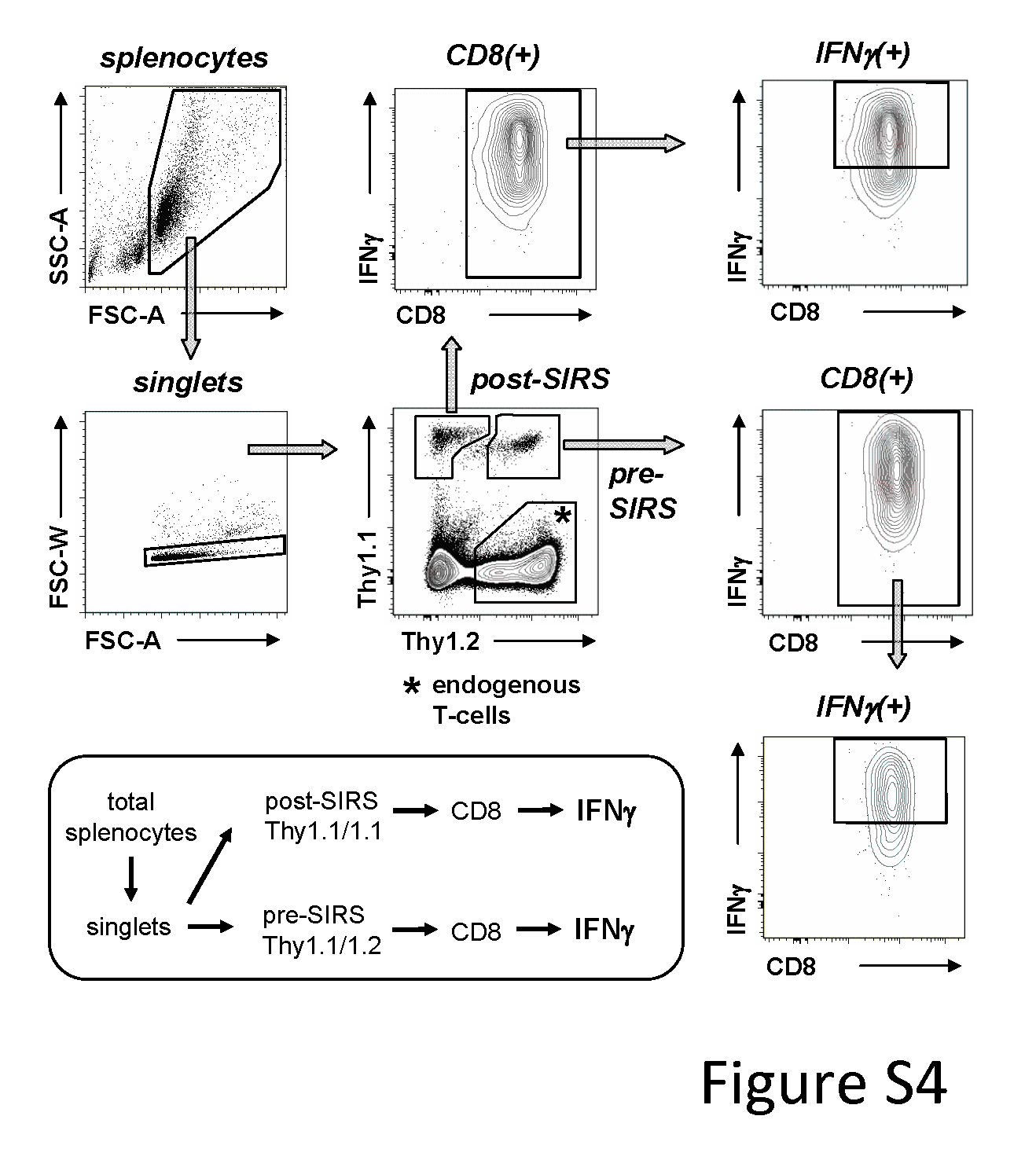

Supplement: S4 Fig — Gating strategy. Representative full gating strategy for adaptively transferred P14 T-cells. Splenic cells were identified via forward scatter (FSC)/side scatter (SSC) blotting followed by singlet gating using FSC-area (A)/FSC-width (W) blotting. Pre-SIRS P14, post-SIRS P14 and endogenous T-cells were discriminated on the basis of their different expression profile of Thy1.1 and Thy1.2 (pre-SIRS: Thy1.1/1.2; post-SIRS: Thy1.1/1.1; endogenous: Thy1.2/1.2). The percentage of IFNγ-expressing (IFNγ+) cells was analysed in CD8+ pre-SIRS and post-SIRS P14 T-cells. Gate for IFNγ+ P14 cells was set judged on baseline IFNγ in non challenged P14 T-cells (see Fig. 3). (TIF) [file pone.0115094.s004.tif]

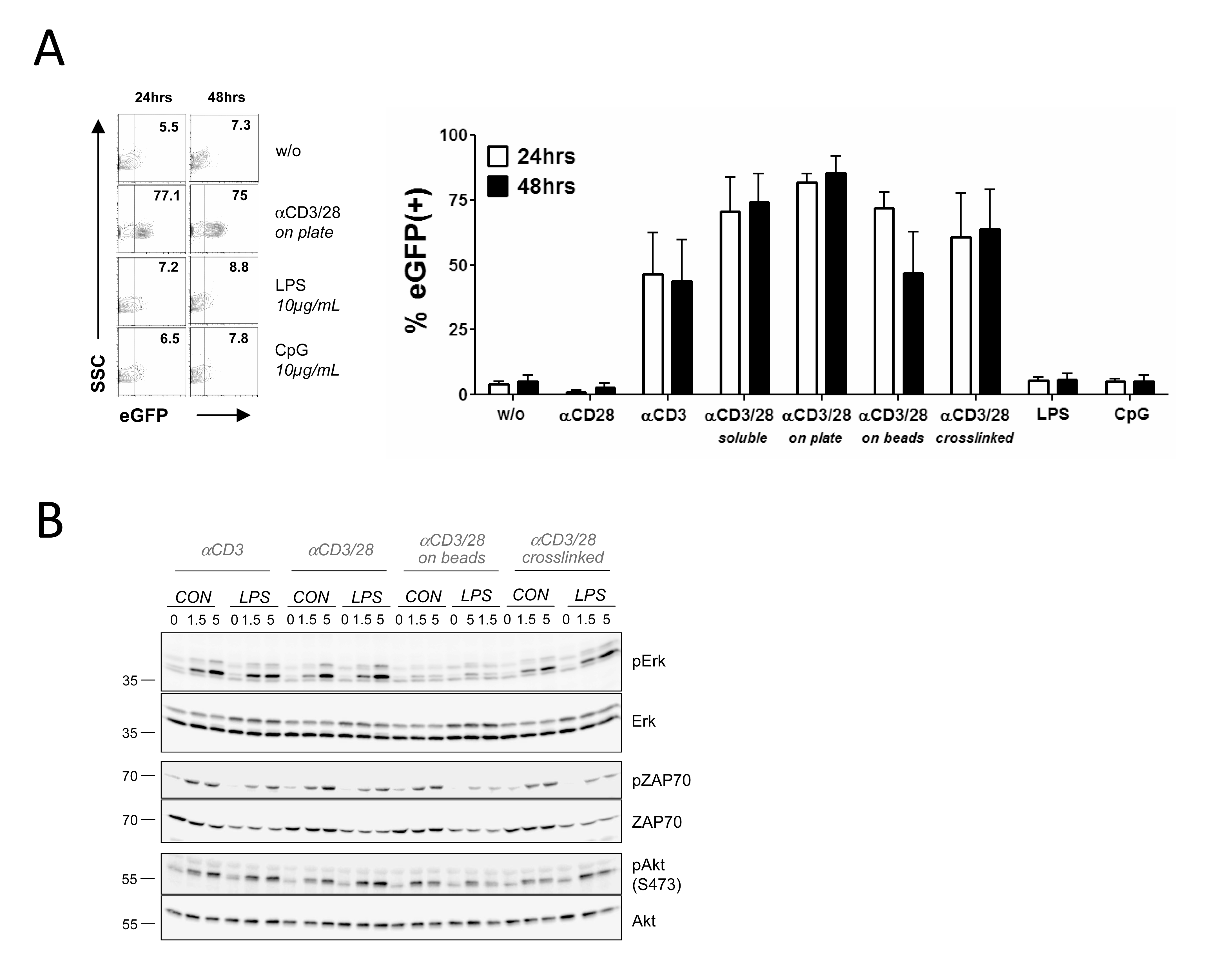

Supplement: S5 Fig — T-cell response to a panel of TCR/co-receptor Abs reflects the requirement for co-stimulation and receptor clustering. (A) Splenic CD4+/CD8+ T-cells purified from transgenic C57BL/6 Tg(Nr4a1-EGFP/cre mice) (a mouse strain expressing EGFP under control of the native Nur77 promotor) were stimulated 24 h and 48 h with a panel of different TCR/co-receptor mAb combinations, 10 µg/ml LPS or 10 µg/ml CpG. EGFP expression as a readout of TCR-dependent Nur77 up-regulation was assessed by flow cytometry. Data are presented as mean + SEM and represent 3-4 independently processed and analysed mice. (B) CD4+/CD8+ T-cells purified from control healthy animals (CON) or from mice 10 days post SIRS/sepsis were stimulated ex vivo with biotinylated CD3ε and/or CD28 mAb administered in solution, either alone or in the presence of the clustering agent streptavidin (crosslinked), or surface-immobilised on latex beads. Cell lysates were subjected to western blot analysis of phosphorylated and total protein levels of Erk, ZAP70 and Akt. Depicted Western blots are representative of several independent experiments. (TIF) [file pone.0115094.s005.tif]

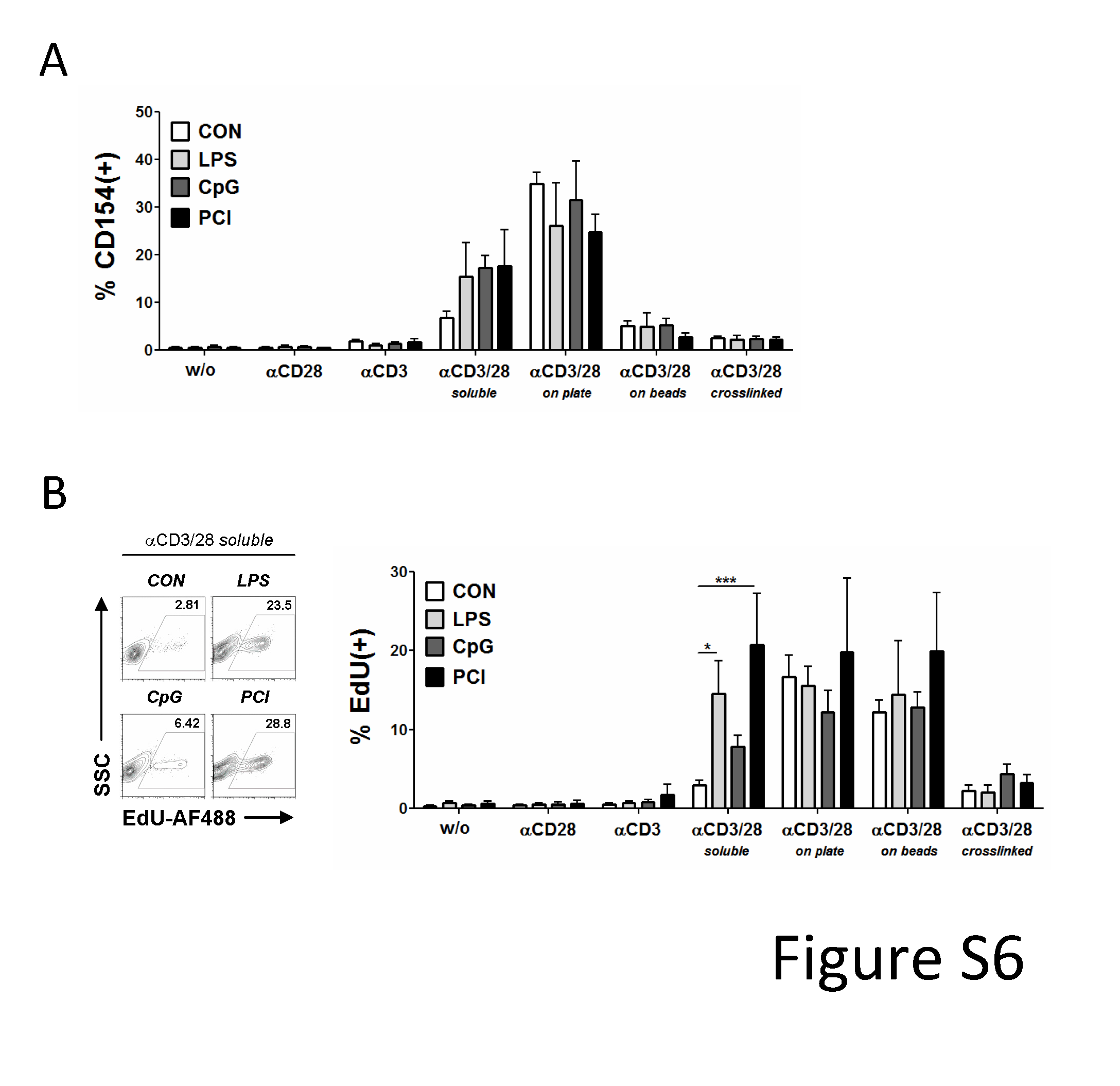

Supplement: S6 Fig — The response of isolated T-cells from post-acute SIRS/sepsis to TCR activation is not compromised. (A) Murine splenic CD4+/CD8+ T-cells purified magnetically 10 days after induction of SIRS/sepsis were stimulated ex vivo with a panel of TCR-triggers. 18 h later surface expression of the activation marker CD154 was assessed with flow cytometry. Data are presented as mean + SEM and represent at least four independent experiments each including at least 4 mice per group. There were no significant differences between experimental groups (One-way ANOVA with post-hoc Bonferroni analysis) (B) 48 h after stimulation DNA synthesis was assessed as a surrogate of cell proliferation by measuring the incorporation of the thymidine analogue 5-ethynyl-2′-deoxyuridine (EdU) into cellular DNA. Data are presented as mean + SEM and represent at least three independent experiments each including at least 4 mice per group. A One-way ANOVA with post-hoc Bonferroni analysis was performed to determine significances (** p≤0.01, ***p≤0.001). Only significant differences among groups are highlighted. (TIF) [file pone.0115094.s006.tif]
